# Pharmacological mechanisms of traditional Chinese medicine in treating gastric cancer: a focus on the ferroptosis regulation

**DOI:** 10.3389/fphar.2025.1700849

**Published:** 2026-01-26

**Authors:** Shanshan Gao, Huijuan Wang

**Affiliations:** 1 First Clinical Medical College, Shandong University of Traditional Chinese Medicine, Jinan, China; 2 Experimental Center, Shandong University of Traditional Chinese Medicine, Jinan, China

**Keywords:** gastric cancer, ferroptosis, traditional Chinese medicine extracts, traditional Chinese medicine formulas, pharmacological mechanism

## Abstract

**Introduction:**

In recent years, the incidence and mortality rates of gastric cancer (GC) have continued to rise, making it a major public health concern that poses a severe threat to human health. Although significant progress has been made in the treatment of GC in recent years, the overall median survival time for patients remains short. This situation is primarily attributed to the high metastatic potential and recurrence rate of tumors, as well as the generally low sensitivity of patients to radiotherapy and chemotherapy. Therefore, the development of safe, effective, and sustainable therapeutic strategies has become an urgent and critical issue in global medical research. Chinese herbal medicine, with its unique advantages such as low cost, minimal risk of drug resistance, and fewer adverse effects, has accumulated extensive clinical experience in the treatment of GC, demonstrating broad application prospects.

**Methods:**

We systematically searched databases including PubMed, Web of Science, Scopus, and ISI using the following keywords: “gastric cancer/tumor,” “natural products,” “natural extracts,” “traditional Chinese medicine extracts,” “traditional Chinese medicine formulas,” and “ferroptosis.” A comprehensive review and analysis of the existing relevant literature were conducted.

**Results:**

Currently, the management of gastric cancer (GC) primarily relies on surgical resection, complemented by a range of therapeutic modalities including radiotherapy, chemotherapy, targeted therapy, and immunotherapy. However, the overall median survival rate for patients with GC remains unsatisfactory. In recent years, accumulating evidence has indicated that Chinese herbal medicine may play a significant role in the treatment of GC through the regulation of ferroptosis. Emerging studies have further demonstrated that Chinese herbal medicine not only induces ferroptosis in tumor cells and suppresses tumor proliferation but also enhances the therapeutic efficacy of radiotherapy, targeted therapy, and immunotherapy, thereby improving overall treatment outcomes. This article reviews recent advances in research on Chinese herbal medicine-mediated modulation of ferroptosis in GC and discusses the potential molecular mechanisms underlying ferroptosis in this malignancy, aiming to provide a theoretical foundation for the development of precise diagnostic approaches and targeted therapeutic strategies.

**Conclusion:**

Studies indicate that Chinese herbal medicines targeting ferroptosis hold promising potential in GC therapy, not only laying a theoretical foundation for elucidating the pathogenesis of GC and establishing ferroptosis-targeted Chinese herbal medicine intervention strategies, but also opening new avenues for the clinical prevention and treatment of GC.

## 1 Introduction

Gastric cancer (GC) represents a common gastrointestinal malignancy that arises from the epithelial and glandular epithelial cells of the gastric mucosa. GC is recognized as the fifth most prevalent ty This substantially heightens the risk of tumor metastasis and augments the likelihood of developing drug resistancepe of cancer worldwide and is also the third leading cause of cancer-related deaths (Johnston and Beckman, 2019). However, due to the absence of distinctive clinical features in the early stages of GC, timely detection poses a significant challenge. Many patients receive a diagnosis only after the tumor has advanced to a later stage (Zhang et al., 2024). This substantially heightens the risk of tumor metastasis and augments the likelihood of developing drug resistance, Furthermore, this results in a 5-year survival rate that merely ranges from 5% to 20% for patients diagnosed with advanced GC (Mamun et al., 2024). Currently, surgical resection is regarded as the primary therapeutic approach for GC. In addition, other adjuvant therapies include chemotherapy, radiotherapy, immunotherapy, and targeted therapy, among others (Wang G. et al., 2024). Despite considerable advancements in prolonging the survival duration of GC patients, more than 70% of patients continue to succumb to the disease (Loe et al., 2021). Therefore, there is an urgent need to identify novel therapeutic targets and develop effective agents for the treatment of GC.

Programmed cell death (PCD) is an actively regulated process of cellular demise governed by genetic factors. It plays a fundamental role in the development, maintenance of homeostasis, and immune defense of organisms (Yuan and Ofengeim, 2024). Ferroptosis, a novel form of programmed cell death (PCD) first introduced by Dixon in 2012 (Dixon et al., 2012), is distinct from other types such as autophagy and apoptosis. Its defining characteristic is the excessive accumulation of iron-dependent lipid peroxides, which ultimately results in damage to the cell membrane (Stockwell et al., 2017). In the field of cytology, ferroptosis is characterized by a reduction in cell volume, preservation of cell membranes devoid of vacuoles, an increase in mitochondrial membrane density, and a reduction or absence of cristae. This process is often accompanied by the rupture of the outer membrane (Doll and Conrad, 2017). The ferroptosis pathway is critically involved in the initiation and progression of GC. From a disease risk perspective, abnormalities in iron metabolism—including anemia, reduced serum ferritin levels, and disorders of iron absorption associated with autoimmune gastritis—have been identified as significant risk factors for GC and other gastrointestinal malignancies (Nomura et al., 2025). Regarding disease progression, the ferroptosis state exhibits a close relationship with the proliferative activity, invasive capacity, and metastatic potential of GC cells (Huang et al., 2021; Chen et al., 2021a). Furthermore, as a promising therapeutic target, ferroptosis inducers have demonstrated their ability to overcome drug resistance in GC cells during clinical studies (Shin et al., 2018). This finding offers new avenues for intervention in the treatment of GC. Overall, these insights underscore the substantial significance of ferroptosis in both the diagnosis and treatment of GC.

Chinese herbal medicine plays a significant role as an adjunct therapy in the field of oncology, characterized by its multi-target effects and low toxicity (Wang et al., 2020). It not only serves as a potential candidate for chemoprevention of GC through mechanisms such as anti-inflammatory, antioxidant activities, and modulation of cell proliferation and differentiation but also effectively synergizes with surgery, radiotherapy, and chemotherapy (Xu et al., 2018; Wang P. et al., 2022). This synergy contributes to improved quality of life for patients, enhanced treatment tolerance, and significant delays in disease progression (Xu et al., 2013). Recent studies have indicated that Chinese herbal medicine possesses unique advantages in the prevention and management of GC and its precancerous lesions primarily by regulating ferroptosis in tumor cells through various pathways and targets. However, the specific mechanisms underlying these effects require further investigation, particularly in terms of transcriptomics, proteomics, metabolomics, and signaling pathways.

In summary, this article examines the application of Chinese herbal medicine in the treatment of GC, systematically investigating its unique mechanisms for regulating ferroptosis through various pathways and targets to intervene in both GC and precancerous lesions. This not only offers a novel integrated perspective on the interplay between Chinese herbal medicine’s anti-gastric cancer mechanisms and ferroptosis regulation but also elucidates core targets and key pathways involved in Chinese herbal medicine’s modulation of ferroptosis. By synthesizing recent basic research evidence, it addresses the existing gap in understanding the mechanistic relationship between the “multi-target action of Chinese herbal medicine” and the “precise regulation of ferroptosis.” Furthermore, the analysis presented herein regarding the anti-gastric cancer potential of active components and formulations of Chinese herbal medicine provides valuable theoretical references for clinical translational research as well as for developing new anti-cancer drugs. Thus, this review underscores its dual significance within academic inquiry and practical application.

## 2 Methodology

**FIGURE 1:**
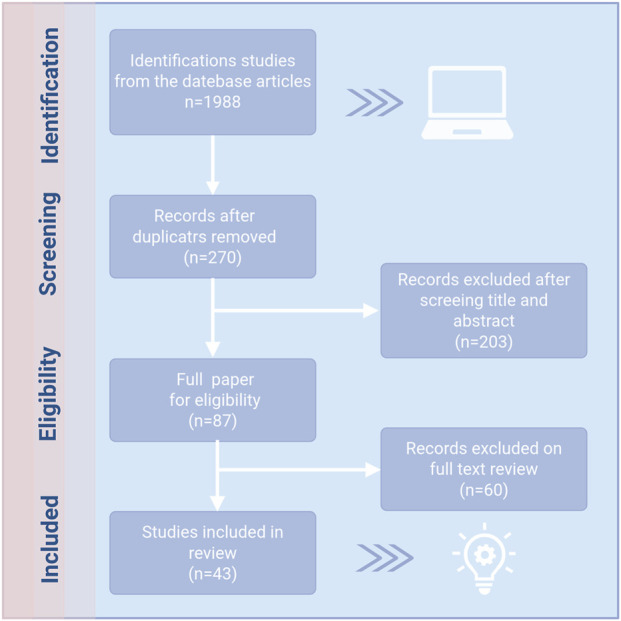
The process of literature retrieval and screening.

To comprehensively investigate the potential role of Chinese herbal medicine in the prevention and treatment of GC through the modulation of ferroptosis in cellular systems, this study implemented a systematic literature search strategy that conforms to the Preferred Reporting Items for Systematic Reviews and Meta-Analyses (PRISMA) guidelines. The search encompassed several authoritative databases, such as PubMed, Web of Science, Scopus, and ISI, among others. The key terms utilized in this study encompass “gastric cancer/tumor,” “natural products,” “natural extracts,” “traditional Chinese medicine extracts,” “Traditional Chinese medicine formulas,” and “ferroptosis.” By systematically employing Boolean logical operators (AND/OR) and methodically integrating keywords, one can enhance both the efficiency and accuracy of information retrieval. During the literature screening phase, two independent researchers conducted a systematic evaluation of the titles, abstracts, and full texts of the identified literature in accordance with pre-established inclusion and exclusion criteria. This procedure guarantees the objectivity and independence of the review process, thereby preventing any potential mutual interference. The inclusion criteria are as follows: (a) original research articles published in English; (b) studies that investigate the pharmacological mechanisms of Chinese herbal medicine in the prevention and treatment of GC through the regulation of ferroptosis and associated signaling pathways, conducted both *in vivo* and *in vitro*. Exclusion criteria are as follows: (a) articles not published in English; (b) review articles; (c) editorial pieces; (d) duplicate publications; and (e) grey literature. After a systematic retrieval process and rigorous screening, a total of 27 studies that fulfilled the inclusion criteria were ultimately selected for further analysis (Figure 1).

## 3 Overview of the ferroptosis

**FIGURE 2:**
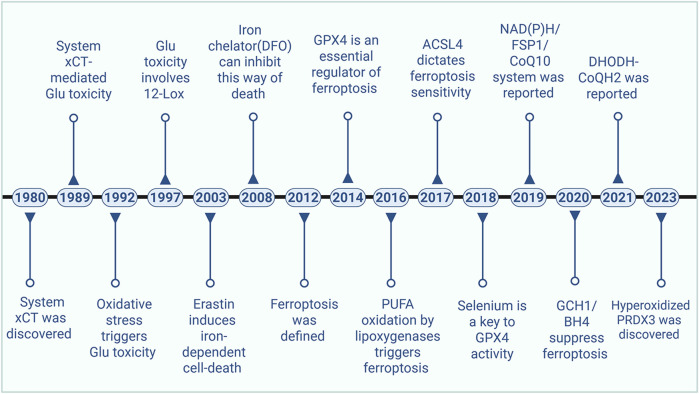
The development history of ferroptosis. ACSL4: acyl-CoA synthetase family member 4, BH4: tetrahydrobiopterin, CoQ10: Coenzyme Q10, DHODH: dihydroorotate dehydrogenase, FSP1: Ferroptosis suppressor protein 1, GCH1: GTP Cyclohydrolase 1, Glu: glutamate, GPX4: Glutathione peroxidase 4, PRDX3: Peroxiredoxin 3, PUFA: Polyunsaturated Fatty Acid.

Although the term “ferroptosis” was first introduced in 2012, distinct forms and features of cell death linked to ferroptosis had been documented well before this designation. In 2003, Dolma et al. (2003) identified a novel compound known as Erastin, which exerted selective cytotoxicity against RAS-expressing cancer cells. However, the mechanism of cell death induced by this compound was markedly distinct from that reported in previous studies: no alterations in nuclear morphology, DNA fragmentation, or caspase activation were observed. Notably, this cell death process could not be abrogated by caspase inhibitors. In 2007, Yagoda et al. (2007) identified that Erastin could induce oxidative cell death by facilitating the release of reactive oxygen species (ROS) from mitochondria. This mechanism is different from conventional forms of cell death, including apoptosis and autophagy. Key characteristics of this process include structural damage to mitochondria and alterations in the permeability of the outer mitochondrial membrane. One year later, Yang et al. further identified that this mode of cell death was associated with iron content (Yang and Stockwell, 2008). In 2012, Dixon et al. (2012) introduced the term “ferroptosis” to describe the cell death of RAS-mutated cancer cells induced by Erastin, based on its distinctive characteristics. To date, ferroptosis has attracted substantial research interest, driving the identification of numerous novel targets and signaling pathways (Figure 2).

## 4 The mechanism of ferroptosis

**FIGURE 3:**
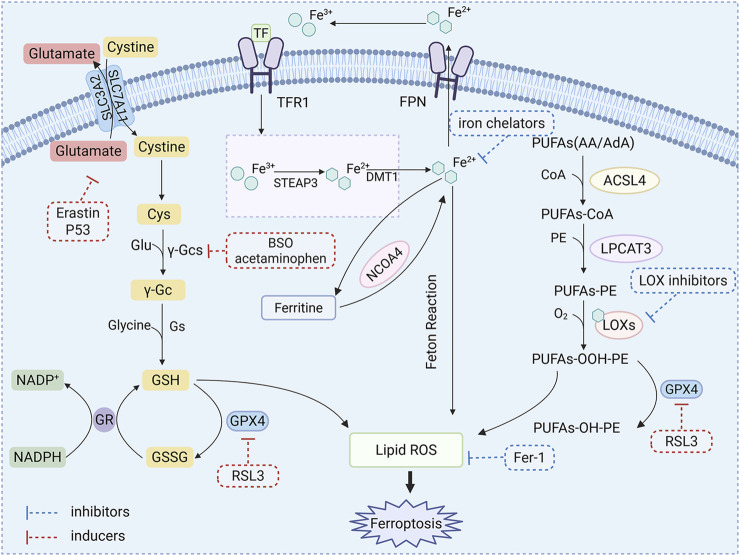
The molecular mechanism of ferroptosis. ACSL4: acyl-CoA synthetase family member 4, CoA: coenzyme A, Cys: cystine, DMT1:divalent metal transporter 1, FPN: ferroportin, γ-GCS: γ-glutamylcysteine synthetase, Glu: glutamate, GS: glutamine synthetase, GPX4: Glutathione peroxidase 4, GSH: glutathione, GSSG: oxidized glutathione, LOX: lipoxygenase, LPCAT3: lysophosphatidylcholine acyltransferase 3, NADPH: nicotinamide adenine dinucleotide phosphate (reduced form), NCOA4: nuclear receptor coactivator 4, PE: Phosphatidylethanolamine, PUFAs: Polyunsaturated Fatty Acids, SLC3A2:solute carrier family 3 member 2, SLC7A11: solute carrier family 7 member 11, STEAP3: six transmembrane epithelial antigen of prostate 3, TF: transferrin, TFR1: transferrin receptor 1.

The mechanism underlying ferroptosis is intricate and is collaboratively regulated by iron metabolism, lipid metabolism, amino acid metabolism, and associated signaling pathways (Figure 3). Among these mechanisms, System Xc^−^ located on the cell membrane, which is composed of solute carrier family 7 member 11 (SLC7A11) and solute carrier family 3 member 2 (SLC3A2) (Bridges et al., 2012), facilitates the exchange of intracellular glutamate for extracellular cystine at a 1:1 ratio (Wu et al., 2019). Cystine undergoes reduction to form cysteine, thereby participating in the synthesis of glutathione (GSH). Glutathione peroxidase 4 (GPX4), which relies on GSH, has the capability to reduce lipid peroxides to lipid alcohols, consequently inhibiting ferroptosis. Furthermore, Erastin can obstruct cystine uptake mediated by System Xc^−^. Depletion of GSH or abnormal expression levels of GPX4 may influence cellular sensitivity to ferroptosis (Dixon et al., 2012). The tumor suppressor gene p53 exerts bidirectional regulation on ferroptosis. It can promote ferroptosis by enhancing the expression of SAT1 through the SAT1/arachidonate 15-lipoxygenase (ALOX15) pathway (Ou et al., 2016) and inhibiting SLC7A11 to activate arachidonate 12-lipoxygenase (ALOX12) (Chu et al., 2019). Conversely, it can also inhibit ferroptosis by promoting GPX4 production via the p21/GSH pathway and blocking DPP4 activity (Tarangelo et al., 2018). The mechanisms underlying p53’s target genes remain to be elucidated. Iron metabolism balance is fundamental to regulatory processes. Fe^3+^ is reduced to Fe^2+^ by duodenal cytochrome B reductase (DCYTB) and subsequently absorbed by divalent metal transporter 1 (DMT1) (Tang et al., 2021). The Fe^2+^ ions are then oxidized back to Fe^3+^, which binds to transferrin (TF). This complex undergoes endocytosis via transferrin receptor 1(TFR1) (Frazer and Anderson, 2014), where it is again reduced to Fe^2+^ within the cell. Iron can be stored in the labile iron pool (LIP) and ferritin, which is composed of FTH1 and FTL subunits. Excessive levels of Fe^2+^ are excreted through ferroportin (FPN). Iron regulatory element-binding protein 2 (IREB2) plays a crucial role in regulating iron metabolism. Additionally, Heat shock protein β-1 (HSPB1) has been shown to decrease iron uptake and lipid peroxidation by inhibiting TFR1, thereby suppressing ferroptosis (Dixon et al., 2012). Notably, iron contributes to lipid peroxidation through the Fenton reaction or acts as a cofactor for lipoxygenase (LOX) and porphyrin oxygenase (POR) (Liu et al., 2022). As the primary driving force of ferroptosis, lipid peroxidation is initiated by the removal of hydrogen atoms from the polyunsaturated fatty acyl groups present in PUFA-PLs. This process generates free radicals that subsequently react to form lipid peroxides (PLOOH) (Conrad and Pratt, 2019). If not reduced by GPX4, these lipid peroxides can accumulate within cells. Phosphatidylethanolamines (PEs) containing arachidonic acid (AA) and adrenic acid (AdA) are critical substrates involved in this pathway (Kagan et al., 2017), These substrates (AA-PE and AdA-PE) are primarily synthesized by acyl-CoA synthetase long-chain family member 4 (ACSL4) and lysophosphatidylcholine acyltransferase 3 (LPCAT3) (Kagan et al., 2017; Chen et al., 2021b). These phospholipids undergo transformations into peroxidation products such as PE-AA-OOH and PE-AdA-OOH through Fenton reactions or lipoxygenase (LOX) catalysis, ultimately inducing ferroptosis. LOX is recognized as a key enzyme facilitating enzymatic lipid peroxidation; thus, knockout of its gene has been shown to inhibit Erastin-induced ferroptosis (Yang et al., 2016).

## 5 Regulatory mechanisms of ferroptosis in GC and precancerous lesions of GC

### 5.1 Ferroptosis and GC

Accumulating evidence has demonstrated that ferroptosis-related molecules (including non-coding RNAs), and exosomes are closely associated with the onset and progression of GC. Furthermore, these findings underscore the significant role of ferroptosis regulation in the prevention and treatment of GC.

#### 5.1.1 Ferroptosis-related molecules in GC

##### 5.1.1.1 GPX4

GPX4 is a key protein in the classical antioxidant system. It catalyzes the reduction of lipid peroxides to harmless lipid alcohols through the action of GSH, thereby interrupting the chain reaction of lipid peroxidation and inhibiting ferroptosis. The catalytic activity of GPX4 is dependent on the availability of GSH. When cysteine is deficient, the synthesis of GSH decreases, which indirectly inhibits downstream GPX4. The impairment of GPX4 function can result in the swift accumulation of lipid peroxides, subsequently initiating ferroptosis (Stockwell et al., 2017). Furthermore, certain compounds or signaling molecules can also directly inhibit GPX4 activity to induce ferroptosis. The dependency of tumor cells on Fe^2+^ often leads to elevated GPX4 expression in GC tumors; GPX4 then inhibits the lipid peroxidation chain reaction, thereby reducing oxidative damage in GC cells. This provides strong evidence for the involvement of ferroptosis mechanisms in the prevention and treatment of GC. Wang Y. et al. (2022) found that activation of the Wnt/β-catenin signaling pathway in GC cells leads to the direct binding of the β-catenin/TCF4 transcriptional complex to the promoter region of GPX4, resulting in enhanced expression of this gene. This mechanism subsequently inhibits ferroptosis in GC cells. A study by Sugezawa et al. (2022) demonstrates that GPX4 exhibits activity across all GC cell lines. Silencing of GPX4 leads to the production of ROS, which in turn suppresses the proliferation of GC cells. Additionally, this research establishes that GPX4 is a significant risk factor affecting the survival of patients with GC.

##### 5.1.1.2 SLC7A11

System Xc-is a crucial component in the regulatory network of ferroptosis. It is a heterodimer composed of two subunits linked by disulfide bonds. Specifically, these subunits are the light chain subunit SLC7A11 and the heavy chain subunit SLC3A2. SLC7A11, a 12-transmembrane channel protein, forms this heterodimeric transporter with SLC3A2, which mediates the uptake of extracellular cystine (Cys) and efflux of intracellular glutamate (Glu). Once inside the cell, Cys is reduced to cysteine (Cys), a precursor for glutathione (GSH) synthesis (Bridges et al., 2012). GSH serves as a cofactor for GPX4, thereby promoting the clearance of lipid peroxidation products by GPX4 and inhibiting cellular ferroptosis. This suggests that SLC7A11 plays a critical role in the mechanisms related to tumor ferroptosis (Koppula et al., 2018). Tumor cells often rely on the high expression of SLC7A11 to maintain GSH levels, thereby resisting oxidative stress and ferroptosis, which in turn promotes proliferation and metastasis. It was found by Jiang et al. (2023) that SLC7A11 expression is significantly higher in GC tissues than in adjacent non-cancerous tissues. Additionally, knockdown of SLC7A11 was shown to inhibit the proliferation, migration, and invasion of GC cells. Further research has confirmed that the silencing of SLC7A11 can inhibit the Phosphatidylinositol 3-Kinase (PI3K)/Protein Kinase B (AKT) signaling pathway, thereby enhancing lipid peroxidation associated with ferroptosis and exerting an antitumor effect. Research has indicated that GDF15, a member of the atypical transforming growth factor β family, plays a crucial role in promoting tumorigenesis. In GC cells (MGC803), silencing GDF15 can reduce the expression of SLC7A11, thereby blocking System Xc- and inducing ferroptosis, which ultimately inhibits the progression of GC (Chen et al., 2020).

##### 5.1.1.3 P53

The expression of the tumor suppressor gene p53 gradually increases with the progression of GC; mutations in its DNA-binding domain, which cause missense mutations in the p53 protein, can confer gain-of-function activities that promote tumor progression (Liu et al., 2020). When p53 binds to the matching site in the 5′ flanking region of the SLC7A11 gene located on chromosome 4q28-31, it inhibits the transcription of SLC7A11. This action blocks System Xc-, thereby affecting intracellular cystine uptake. Consequently, this inhibition suppresses downstream GSH synthesis and GPX4 activity, preventing GSH-mediated reduction of lipid peroxides and ultimately inducing ferroptosis (Jiang et al., 2015). In addition to regulating ferroptosis via the p53/SLC7A11/GPX4 axis, p53 can also modulate this process through another key pathway involving spermidine/spermine N1-acetyltransferase 1 (SAT1), a crucial regulatory factor in polyamine metabolism. As a transcriptional target of p53, under ROS-induced oxidative stress, p53-mediated activation of SAT1 expression can lead to lipid peroxidation and sensitize cells to ferroptosis (Ou et al., 2016). Furthermore, the induction of SAT1 is closely associated with the activity of ALOX15. The ferroptosis induced by SAT1 is abolished in the presence of specific inhibitors targeting ALOX15 (Ma et al., 2022). Therefore, p53 can regulate ALOX15 by activating SAT1, thereby promoting lipid peroxidation in cells and inducing ferroptosis.

##### 5.1.1.4 P62

The P62 gene possesses the capacity to inhibit programmed cell death (PCD) in tumor cells, thereby complicating their elimination. It plays a significant role during the progression of tumors (Wang et al., 2021). The Kelch-like ECH-associated protein 1 (Keap1)/Nuclear factor erythroid 2-related factor 2 (Nrf2) pathway is a crucial mechanism for collective antioxidant defense. Under homeostatic conditions, Keap1 forms a ubiquitin E3 ligase complex that targets Nrf2 for ubiquitination and subsequent degradation via the proteasomal system. When ROS accumulate within the cell, the cysteine residues of Keap1 undergo modifications, leading to a reduction in its E3 ubiquitin ligase activity. Consequently, Nrf2 accumulates and translocates to the nucleus (Baird and Yamamoto, 2020), where it transcriptionally induces p62 expression. Phosphorylation of p62 enhances its binding affinity to Keap1, specifically at the Ser403 and Ser351 sites, thereby disrupting the Nrf2-Keap1 complex and triggering Nrf2 activation. Consequently, downstream antioxidant response elements are activated, increasing the production of antioxidant proteins while reducing ROS. This regulation affects the expression of ferroptosis-related factors, including GPX4, SLC7A11, and heme oxygenase-1 (HO-1), thereby inhibiting ferroptosis (Wang et al., 2021).

Nrf2 is primarily expressed in the nuclei of GC cells, with significantly lower expression in the cytoplasm. Functionally, reducing Nrf2 levels can inhibit the efflux activity of chemotherapeutic agents and decrease the resistance of GC cells to these drugs of GC cells to chemotherapeutic agents (Jeddi et al., 2018). Wang T. et al. (2022) utilized gene transfection techniques to induce the silencing of a potential transforming growth factor β binding protein (TGFβBP) gene. This silencing led to increased Keap1 expression, decreased p62 levels, and subsequent inhibition of the antioxidant transcription factor Nrf2, thereby activating ferroptosis in GC cells. However, when Nrf2 activity was restored, ferroptosis was suppressed, promoting the proliferation of GC cells.

#### 5.1.2 Ferroptosis-related non-coding RNAs in GC

##### 5.1.2.1 miRNA

MicroRNA (miRNA) can bind to its effector complex, the RNA-induced silencing complex (RISC), achieving complete or partial complementarity with the 3′ untranslated region of mRNA. This interaction can lead to direct cleavage of the mRNA or inhibition of its translation, thereby inducing the repression of target genes (Michlewski and Cáceres, 2019). Ni et al. (2021) utilized gene chip sequencing to discover that miR-375 can target and inhibit SLC7A11, resulting in a reduction in intracellular cystine uptake, which impairs the synthesis of GSH. Consequently, when miR-375 is overexpressed, it leads to the accumulation of lipid ROS, triggering ferroptosis and inducing oxidative damage in GC cells while diminishing tumor stemness. Shao et al. (2023) discovered that the silencing of miR-221-3p upregulates the expression of its target gene, the transcription factor activating transcription factor 3 (ATF3). ATF3 strongly binds to the promoter of SLC7A11 and regulates its transcription, thereby inhibiting GPX4 activity and inducing ferroptosis in GC cells.

##### 5.1.2.2 lncRNA

Long non-coding RNAs (lncRNAs) play crucial roles in GC by regulating ferroptosis, a process closely associated with cancer progression and therapeutic response. lncRNA denotes a category of non-coding RNA molecules that are characterized by their length, exceeding 200 nucleotides. These lncRNAs can interact with DNA, chromatin-modifying complexes, and associated transcriptional regulatory factors to modulate gene expression within the nucleus. Furthermore, cytoplasmic lncRNAs may function as “molecular sponges,” regulating the expression of target mRNAs (Bridges et al., 2021). Wei et al. (2021), in a study focusing on GC prognosis employed univariate and multivariate Cox regression analyses to identify seven ferroptosis-related lncRNAs from the Cancer Genome Atlas. They discovered that Lnc-AP003392.1, AC245041.2, AP001271.1, and BOLA3-AS1 exhibited differential expression between GC cells and normal cells, indicating that these lncRNAs may influence the prognosis of GC by inducing ferroptosis. Yao et al. (2021) discovered that the long non-coding RNAs A2M-AS1, C2orf27A, and ZNF667-AS1 can regulate ferroptosis-related genes such as GPX4. This regulation influences the activation of CD4^+^ T cells, thereby enhancing the efficacy of immunotherapy for GC. Additionally, hypoxia has been found to trigger peritoneal metastasis in GC cells. Lin et al. (2022) discovered that lncRNA-PMAN is highly expressed in peritoneal metastasis due to elevated levels of hypoxia-inducible factor-1α. This upregulation enhances the expression of SLC7A11 and GSH, while reducing the accumulation of ROS and Fe3+, thereby inhibiting ferroptosis in GC cells.

##### 5.1.2.3 circRNA

Circular RNA (circRNA) is produced through the back-splicing of precursor mRNA, which can consist of exons or introns. It has a covalently closed circular structure, lacking 5′-3′ polarity and a polyadenylated tail. This unique configuration allows circRNA to resist degradation by RNA exonucleases, contributing to its relatively stable existence in eukaryotic organisms (Chen L. et al., 2021). The circRNA associated with ferroptosis exhibits differential expression in GC cells, suggesting its potential as a novel molecular target for the prediction and diagnosis of GC. Jiang et al. (2022) discovered that the overexpression of circ0000190 can function as a molecular sponge, specifically targeting and binding to miR-382-5p, thereby promoting ferroptosis in GC cells. Liu J. et al. (2023) circRPPH1 acts as a molecular sponge for miR-375, thereby relieving the inhibitory effect of miR-375 on SLC7A11, enhancing SLC7A11 expression and inhibiting ferroptosis in GC cells. Gao and Wang (2023) discovered that dexmedetomidine can target circ0008035 to enhance the expression of E2F transcription factor 3, subsequently reducing the activities of GSH, GPX4, and SLC7A11. This leads to the accumulation of ROS and induces ferroptosis in GC cells.

#### 5.1.3 Ferroptosis-related exosomes in GC

Exosomes regulate cellular ferroptosis through pathways involving ferritin metabolism, lipid metabolism, and amino acid metabolism. Zhang et al. (2020) focusing on cancer-associated fibroblasts (CAFs)-derived exosomes, discovered that exosomal miR-522 derived from cancer-associated fibroblasts (CAFs) within the tumor microenvironment inhibits ferroptosis in GC cells by targeting and suppressing ALOX 15, thereby blocking ROS accumulation. Additionally, ubiquitin-specific protease 7 (USP7) promotes miR-522 secretion from CAFs by deubiquitinating heterogeneous nuclear ribonucleoprotein A1 (hnRNPA1). Silencing USP7 or hnRNPA1 results in a reduction of miR-522 levels, ultimately leading to increased cellular ferroptosis. Zhang H. et al. (2021) in a study on GC cell-secreted exosomes, found that exosomal lncFERO secreted by GC cells can directly bind to Stearoyl-CoA Desaturase 1(SCD1) mRNA. With the assistance of hnRNPA1, this interaction promotes SCD1 expression and reduces polyunsaturated fatty acid (PUFA) levels, thereby inhibiting ferroptosis and alleviating GC cell chemoresistance. Qu et al. (2023) reported that exosomal lncRNA DACT3-AS1 derived from CAFs targets the miR-181a-5p/Sirtuin 1 (SIRT1) axis, thereby inhibiting the proliferation, migration, and invasion of GC cells. Additionally, lncRNA DACT3-AS1 enhances the sensitivity of GC cells to oxaliplatin and suppresses drug resistance through SIRT1-mediated ferroptosis.

### 5.2 Ferroptosis and precancerous lesions of GC

Currently, the relationship between ferroptosis and precancerous lesions of GC remains not yet fully understood. In the various stages of the Correa cascade model, ferroptosis appears to play distinct roles. In a rat model of chronic atrophic gastritis, the expression levels of ferroptosis marker proteins GPX4 and ferritin heavy chain (FTH) were found to be reduced, while the lipid peroxidation product 4-HNE was elevated. This indicates that during the stage of atrophic gastritis, ferroptosis is in an activated state. Inhibiting ferroptosis has been suggested to prevent the progression of chronic atrophic gastritis (Zhao et al., 2023). Chronic atrophic gastritis in animal models is characterized by a hypoxic gastric mucosal microenvironment, mitochondrial dysfunction, and ROS accumulation. These pathological changes collectively enhance the sensitivity of gastric mucosal cells to ferroptosis (Wu et al., 2019). Due to the limited research on ferroptosis in precancerous lesions of GC, the specific mechanisms underlying different states of ferroptosis during the transition from precancerous lesions to GC require further investigation in the future. This will provide new strategies for the prevention and treatment of precancerous lesions associated with GC.

Furthermore, research has reported the differential genes associated with ferroptosis in precancerous lesions of GC and their underlying mechanisms. The genes associated with ferroptosis exhibit a significant correlation with intestinal epithelial metaplasia in precancerous lesions of GC. The study Song et al. (2023) identified 17 differentially expressed ferroptosis-related genes in intestinal epithelial metaplasia. Utilizing Cytoscape software, they further pinpointed four central genes: PTGS2, HMOX1, IFNG, and NOS2. The diagnostic value of HMOX1 was validated through ROC analysis and qRT-PCR methods, suggesting that HMOX1 may serve as a potential biomarker and therapeutic target for the diagnosis of intestinal epithelial metaplasia. Kuang et al. (2023) identified 23 differentially expressed genes associated with ferroptosis in precancerous lesions of GC. They further validated four central genes: MYB, CYB5R1, LIFR, and DPP4. The regulatory mechanism of non-coding RNA for these central genes was found to be LINC01002/miR-150-5p/central genes (MYB, CYB5R1, LIFR, DPP4).

Currently, the role of ferroptosis in the progression of precancerous lesions of GC has been less extensively investigated compared to its role in established GC. The investigation of the mechanisms underlying the transformation from precancerous lesions to GC, using “ferroptosis” as a focal point, is crucial for the prevention of GC. The infection of *Helicobacter pylori* is a significant factor in the progression from gastric precancerous lesions to cancer. Further research is needed to clarify its relationship with gastric precancerous lesions and iron deficiency. Additionally, it is essential to actively explore the potential mechanisms that may inhibit the advancement of the “bacteria-inflammation-cancer” pathway associated with gastric precancerous conditions.

## 6 Traditional Chinese medicine extracts prevent and treat GC by regulating ferroptosis

**FIGURE 4:**
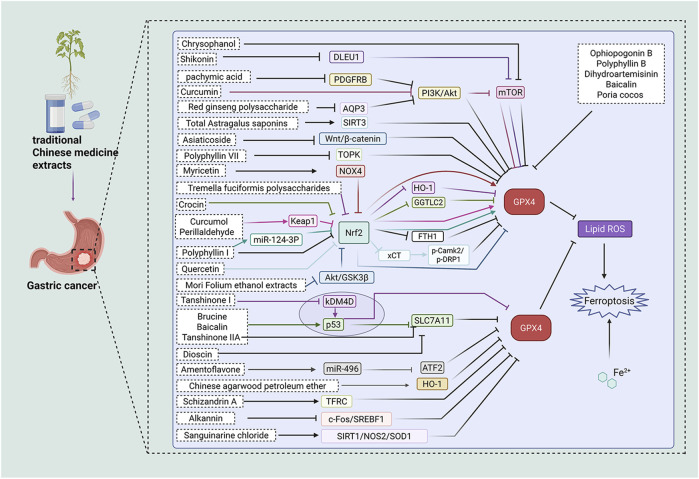
Pharmacological mechanisms of traditional Chinese medicine extracts targeting ferroptosis in the treatment of gastric cancer. ATF2: activating transcription factor 2, ATG5: autophagy related 5, Akt: protein kinase B, ALOX12: arachidonate 12-lipoxygenase, AQP3: aquaporin 3, c-Fos: cellular proto-oncogene fos, DLEU1:deleted in lymphocytic leukemia 1, FTH1: ferritin heavy chain 1, GGTLC2: gamma-glutamyltransferase light chain 2, GPX4: Glutathione peroxidase 4, GSH: glutathione, GSK3β: glycogen synthase kinase 3β, HO-1: heme oxygenase-1, Keap1: Kelch-like ECH-associated protein 1, KDM4D: lysine-specific demethylase 4D, LC3B: Microtubule-associated protein 1 light chain 3 beta, mTOR: mammalian target of rapamycin, NF‐κB: Nuclear Factor kappa-light-chain-enhancer of Activated B Cells, NRF2: Nuclear factor erythroid 2-related factor 2, NOS2: nitric oxide synthase 2, NOX4: NADPH oxidase 4, NQO1: NAD(P)H:quinone oxidoreductase 1, p-CaMKII: phosphorylated calcium/calmodulin-dependent protein kinase II, PDGFRB: platelet-derived growth factor receptor beta, p-DRP1: phosphorylated dynamin-related protein 1, PI3K: Phosphatidylinositol 3-Kinase, SIRT3: sirtuin 3, SLC1A5: solute carrier family 1 member 5, SIRT1: Sirtuin 1, SREBF1: sterol regulatory element-binding factor 1, SLC3A2: solute carrier family 3 member 2, SLC7A11: solute carrier family 7 member 11, SOD1: superoxide dismutase 1, TFR1: transferrin receptor 1, TOPK: tumor protein kinase, ULK1: Unc-51 like autophagy activating kinase 1.

Currently, surgical resection remains the principal treatment modality for GC, typically employed in conjunction with chemotherapy or radiotherapy (Engstrand and Graham, 2020). Immunotherapy and targeted therapies represent innovative treatment modalities currently under investigation, with the potential to enhance patient prognosis (Bessède and Mégraud, 2022). However, for patients with advanced GC, particularly patients with GC that, whose condition has progressed to late stages-the therapeutic outcomes continue to be unsatisfactory. Extensive research indicates that ferroptosis is a pivotal factor in the onset, progression, treatment, and prognosis of GC (Yang et al., 2022). The dysregulation or targeted modulation of ferroptosis-related signaling pathways related to ferroptosis have the potential to reverse resistance to chemotherapy. The study conducted by Zhang et al. (2020) indicates that the promotion of ferritin accumulation may promote chemotherapy resistance in GC. These conflicting findings suggest that the role of ferritin in GC chemotherapy sensitivity is context-dependent, possibly related to the specific stage of GC or the type of chemotherapeutic agents used. Conversely, another study suggests that enhancing ferritin levels can restore the sensitivity of GC to chemotherapy (Ouyang et al., 2022). Traditional Chinese medicine extracts have been shown to target multiple pathways in GC prevention and treatment. They are cost-effective, relatively non-toxic, and readily accessible. Therefore, Traditional Chinese medicine extracts may represent a novel therapeutic approach for GC by modulating ferroptosis in GC cells (Figure 4; Table 1).

**TABLE 1:** Pharmacological mechanisms of traditional Chinese medicine extracts targeting ferroptosis in the treatment of gastric cancer.

Classification	Compounds	Source	Molecular formula	Chemical structure	Dosage of drugs used	Cell lines or animal type	Main indicators	Mechanisms	References
Saponins	Total Astragalus saponins (TAS)	*Astragalus membranaceus* (Fisch.) Bunge	N/A	N/A	50 μg/mL,100 μg/mL, 200 μg/mL	SGC-7901	SIRT3↑, SLC7A11 and GPX4↓, ACSL4↑, MDA, LDH, Fe^2+^, and ROS↑	TAS upregulates SIRT3 to promote the ferroptosis of SGC-7901 cells.	Zou et al. (2025)
	Polyphyllin VII (PPVII)	*Paris polyphylla* Sm	C_51_ H_84_ O_22_	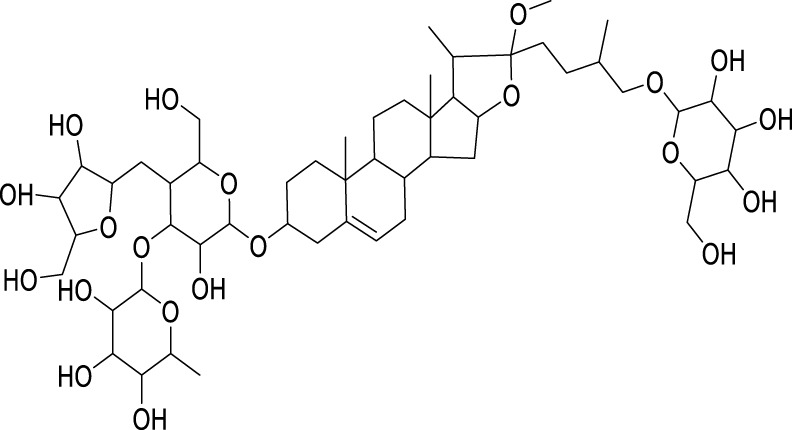	1.2μM, 1.4μM, 1.6 μM	AGS and NCI-N87	TOPK and S757-ULK1↓, phosphorylation level of S555-ULK1↑, FTH1↓, Fe^2+^, MDA, and ROS↑	PPVII activates autophagic ferroptosis by directly targeting and inhibiting TOPK.	Xiang et al. (2023)
	Polyphyllin I (PPI)	*Paris polyphylla* Sm	C_44_ H_70_ O_16_	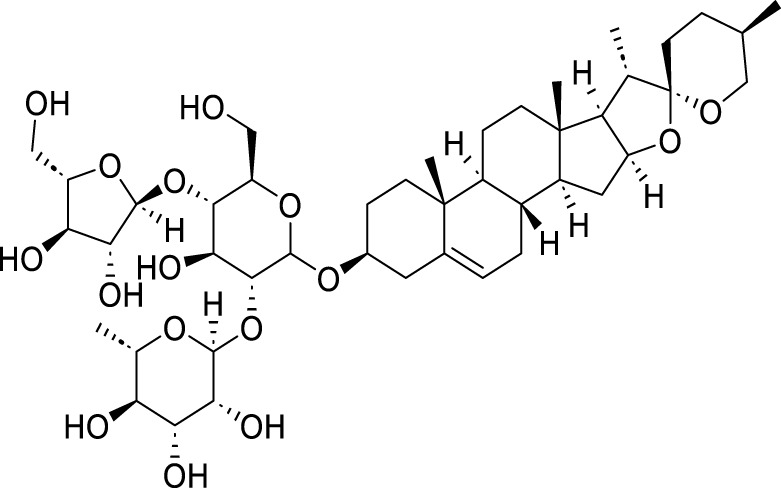	In vitro: 1μM, 2μM, 4 μM In vivo: 30 mg/kg	BALB/c nude mice AGS cells or MKN-45 cells	NRF2 and FTH1↓, ROS, lipid peroxides and ferrous ions↑	PPI induces ferroptosis in BALB/c nude mice AGS cells or MKN-45 cells by regulating the NRF2/FTH1 pathway	Zheng et al. (2023a)
					3 mg/kg	BALB/c nude mice	miR-124-3p↑, NRF 2↓, ROS and Fe^2+^↑	PPI induces ferroptosis in AGS and MKN-45 cells through the modulation of the miR-124-3p/NRF2 signaling axis	Zheng et al. (2023b)
	Polyphyllin B(PB)	*Paris polyphylla* Sm	C_51_ H_82_ O_20_	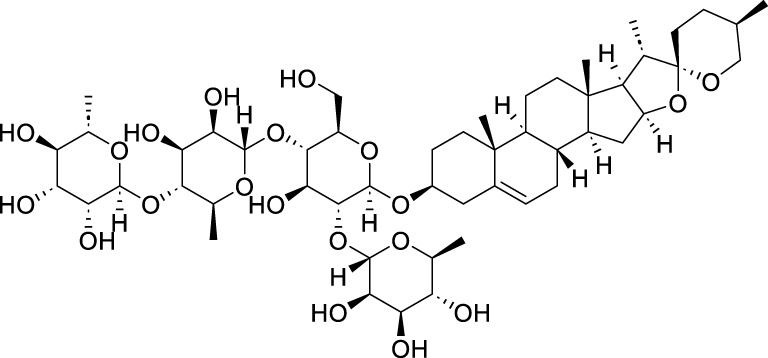	0.5μM, 1μM, 2 μM	MKN-1 and NUGC-3 cells	TFRC, LC3B, NCOA4, and FTH1↑, lipid peroxidation and Fe^2+^↑, GPX 4↓,	PB can induce ferroptosis by downregulating the expression of GPX4 in MKN-1 and NUGC-3 cells	Hu et al. (2023)
	Ophiopogonin B (OP-B)	*Ophiopogon japonicus* (L. f.) Ker Gawl	C_39_H_62_O_12_	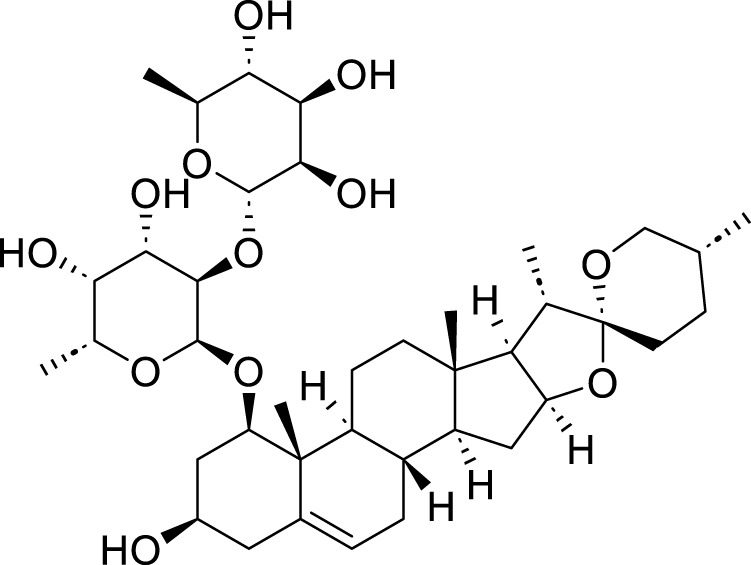	10μM, 20 μM	AGS and NCI-N87	GPX4 and xCT↓, Fe^2+^, MDA, and ROS↑	OP-B induces ferroptosis in AGS and NCI-N87 cells by blocking the GPX4/xCT system	Zhang et al. (2022)
	Asiaticoside (AC)	*Centella asiatica* (L.) Urb	C_48_H_78_O_19_	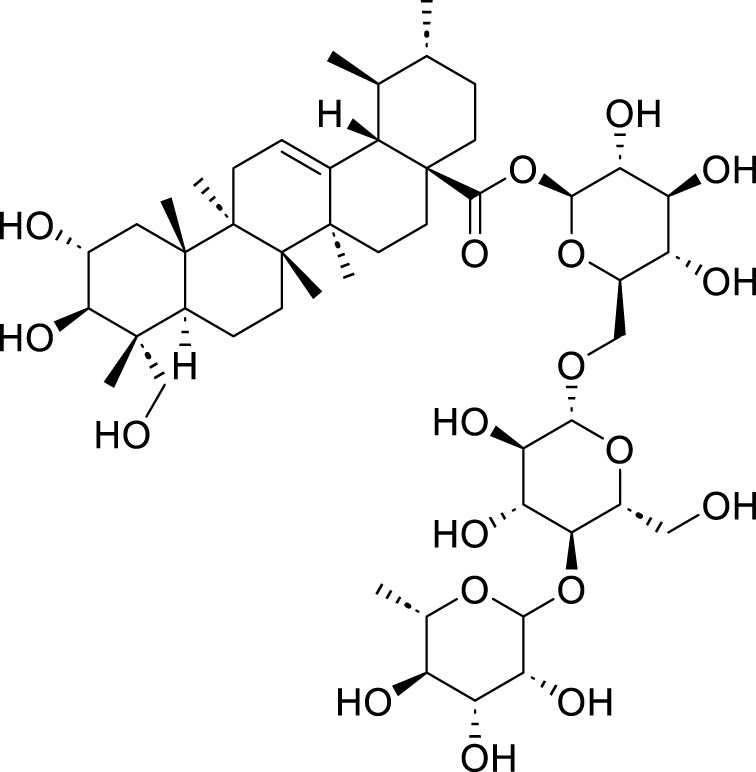	1μM, 2μM, 4 μM	AGS and HGC27	β-catenin, β-catenin, p-GSK3β/GSK3β, c-Myc, and cyclin D1↓, GPX4, SLC7A11, and GSH↓, Fe^2+^ ↑, ROS↑	AC enhanced ferroptosis and repressed immune escape by downregulating the Wnt/β-catenin signaling in AGS and HGC27 cells.	Ye et al. (2024)
Terpenoids	Tanshinone IIA (Tan IIA)	*Salvia miltiorrhiza* Bunge	C_19_H_18_O_3_	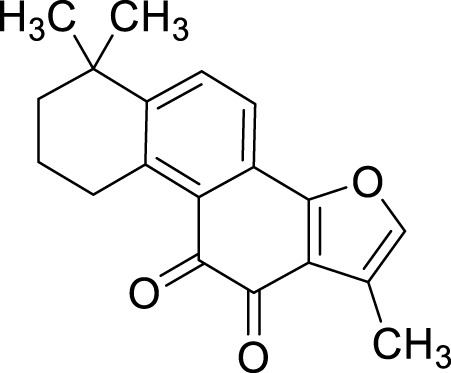	2μM, 4 μM	BGC-823 and NCI-H87 cells	p53↑, GSH↓, xCT↓, lipid peroxidation↑, PTGS2↑, Chac1↑, ROS↑	Tan IIA induces ferroptosis via p53-mediated SLC7A11 downregulation in BGC-823 and NCI-H87 gastric cancer cells	Guan et al. (2020)
					250 μM	SGC-7901 and BGC-823	GSH↓, SLC7A11↓, lipid peroxidation level↑, OCT3/4 ALDH1A1, and CD44↓	Tanshinone IIA suppressed the gastric cancer cell stemness by a SLC7A11-dependent ferroptosis	Ni et al. (2022)
	Tanshinone I (Tan I)	*Salvia miltiorrhiza* Bunge	C_18_H_12_O_3_	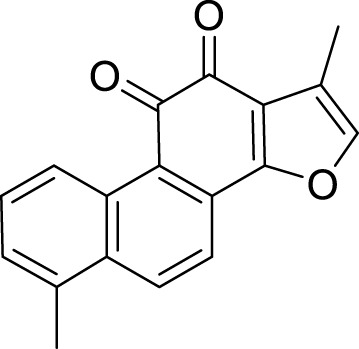	1μM, 3μM, 9 μM	AGS and HGC27	KDM4D↓, p53↑, ACSL4, TFR1, MDA↑, Fe^2+^ ↑, GSH, GPX4, SLC7A11, and FTH1↓	Tan I induced ferroptosis inhibition in AGS and HGC27 cells by regulating the KDM4D/p53 pathway	Xia et al. (2023)
	pachymic acid (PA)	*Poria cocos* (PC)	C_3_H_52_O _5_	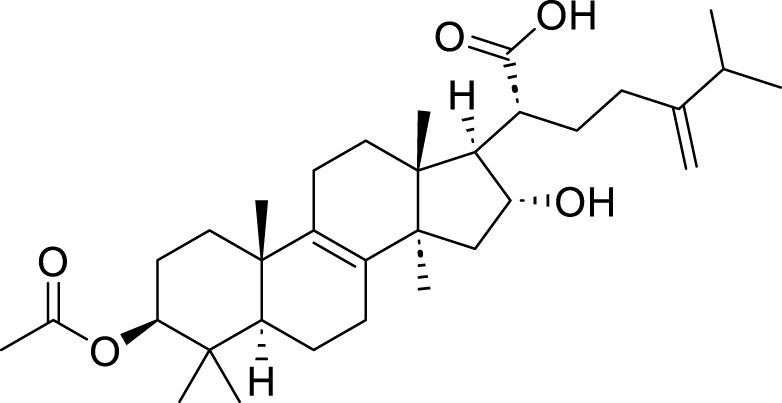	10μM, 20μM, 40 μM	SGC-7901 and AGS	PDGFRB↓, PI3K/Akt↓, GSH, SLC7A11, GPX4↓, MDA, Fe^2+^, and ROS↑	PA induces ferroptosis in SGC-7901 and AGS cells by modulating the PI3K/Akt signalling pathway mediated by PDGFRB.	Nie et al. (2024)
	Curcumol (CUR)	*Curcuma phaeocaulis* Valeton and *Curcuma longa* L	C_15_ H_24_ O_2_	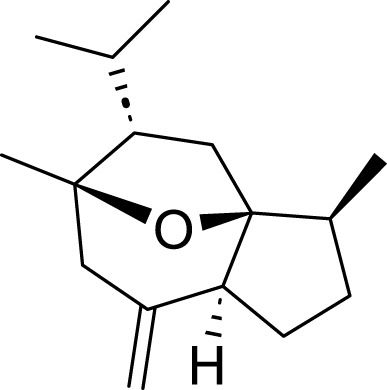	10μM, 25μM, 50μM, 100μM, 125 μM 150 μM	MKN 45/DDP and AGS/DDP	Keap1↑, NRF2, p-p62, NQO1, and GPX4↓ MDA, Fe^2+^, and ROS↑	CUR and CDDP induced ferroptosis in MKN 45/DDP and AGS/DDP cells by regulating the ROS and GSH systems, possibly through modulation of the P62/KEAP1/NRF2 signaling pathway.	Feng et al. (2024)
	Dioscin	*Paris polyphylla* Sm	C_45_H_72_O_16_	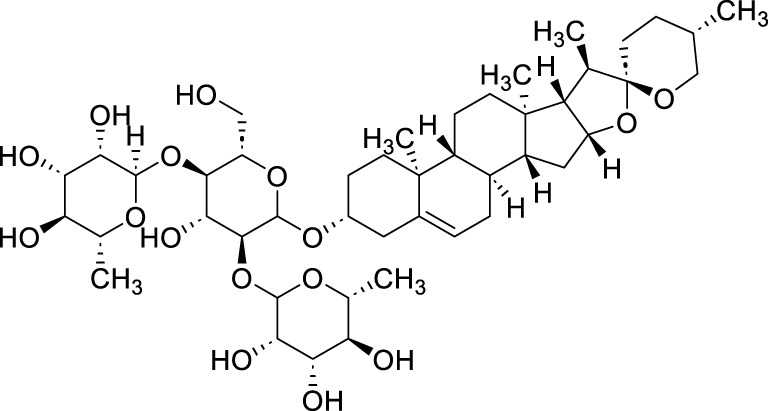	6μM, 8μM, 10μM, 14μM, 16 μM 18 μM	HGC-27 and AGS cells	SLC7A11↓, GPX4↓, ROS↑, MDA↑, GSH↓	Dioscin can induce ferroptosis in HGC-27 and AGS cells through modulation of the SLC7A11/GPX4 signaling axis	Lu et al. (2025)
	Corosolic acid (CA)	*Actinidia chinensis* Planch	C_30_H_48_O_4_	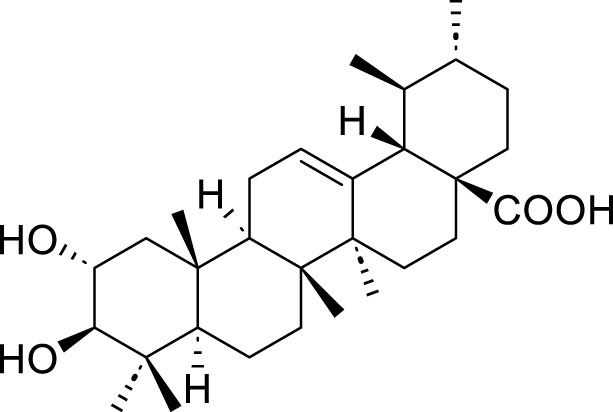	5μM, 10μM, 20μM, 4 μM	AGS-CR cells	GSH↓, GPX4↓, ROS↑, MDA↑	CA increases cisplatin sensitivity in GC by regulating Gpx4-dependent ferroptosis	Lin et al. (2025)
	Perillaldehyde (PAH)	*Perilla frutescens* (L.) Britton	C_10_H_14_O	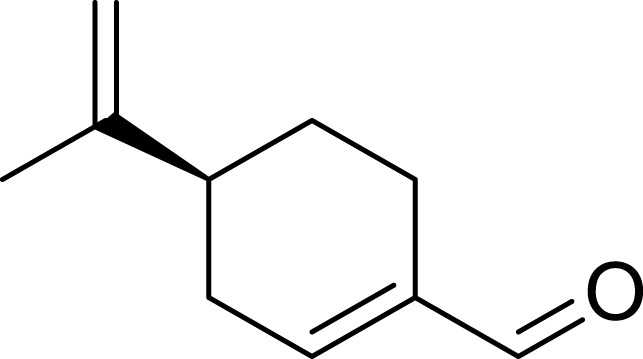	50 μmol/L, 100 μmol/L, 150 μmol/L, 200 μmol/L, 250 μmol/L, 300 μmol/L, 350 μmol/L, 400 μmol/L	AGS and HGC27 cells	NRF2↑, Keap1↑, P62↓, GPX4 and SLC7A11↓, GSH↓, GPX4↓, ROS↑, MDA↑	PAH modulates ferroptosis in AGS and HGC27 cells through the activation of the P62/Keap1/NRF2 antioxidant signaling pathway	Wang et al. (2025a)
	Dihydroartemisinin (DHA)	Artemisia caruifolia Buch.-Ham. ex Roxb	C_15_H_24_O	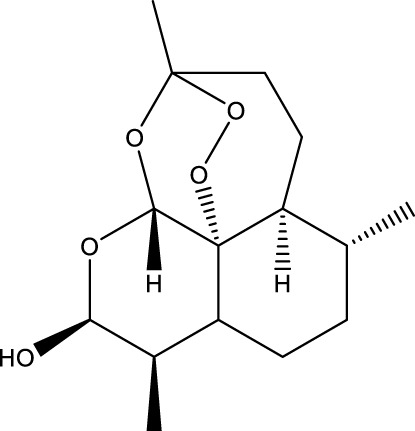	25 μM	SGC-7901 and HGC-27 cells	GPX4↓, ROS↑, MDA↑	DHA acts in combination with DDP to induce ferroptosis in SGC-7901 and HGC-27 cells by inhibiting GPX4	Wang et al. (2025b)
Flavonoids	Quercetin (Quer)	various plants	C_15_H_10_O_7_	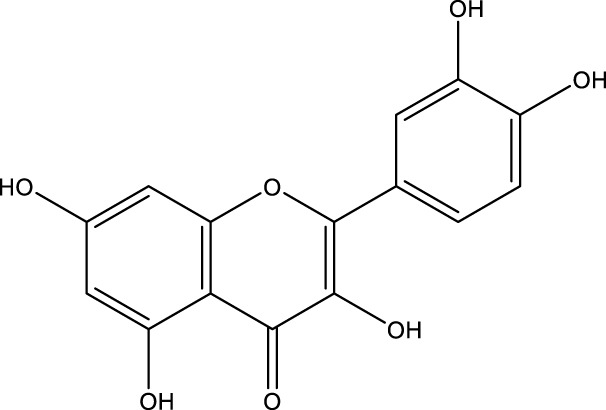	20µM, 40µM, 80µM, 160µM, 320 µM 640 µM	AGS cells	NRF2, xCT↓, SLC1A5↓, GPX4↓, GSH↓, p-Camk2, p-DRP1, MDA, and ROS↑	Quer targets SLC1A5 in AGS cells, inhibiting the NRF2/xCT pathway, activating the p-Camk2/p-DRP1 pathway, and promoting ferroptosis	Ding et al. (2024)
					40 μM	AGS and MKN45 cells, as well as BALB/c mice	ATG5, LC3B and Beclin-1↑, TFR1, GPX4, SLC7A11, and GSH↓, ROS, MDA↑	Quer mediates ferroptosis in AGS and MKN45 cells, as well as BALB/c mice is associated with ATG5-mediated ferritinophagy	Huang et al. (2024a)
	Baicalin	*Scutellaria baicalensis* Georgi	C_21_H_18_O_11_	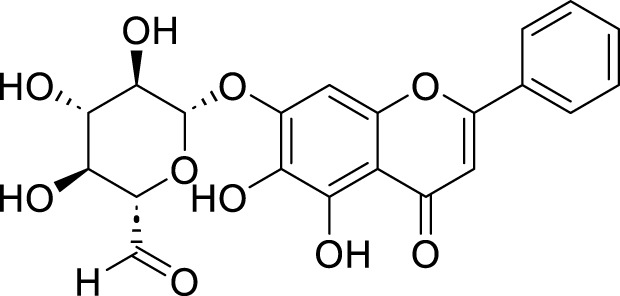	30 μg/mL, 60 μg/mL, 90 μg/mL, 120 μg/mL, and 150 μg/mL	AGS and SGC-7901 cells	TFR1, NOX1, COX2, and GPX4↓, ROS↑	Baicalin inhibits GC and enhances 5-Fu by promoting ROS-related ferroptosis in AGS and SGC-7901 cells	Yuan et al. (2023)
					100µM, 150µM, 200µM, 250 µM	drug-resistant HGC27/L cell line	p53↑, FTH1↓, SLC7A11↓, GPX4↓, TFRC, MDA, and ROS↑	Baicalin could enhance the chemotherapy sensitivity of resistant gastric cancer cells HGC-27/L by activating ferroptosis through upregulation of SLC7A11/GPX4/ROS mediated by tumor suppressor gene p53	Shao et al. (2024)
	Myricetin	*Morella rubra* Lour	C_15_​H_10_​O_8_​	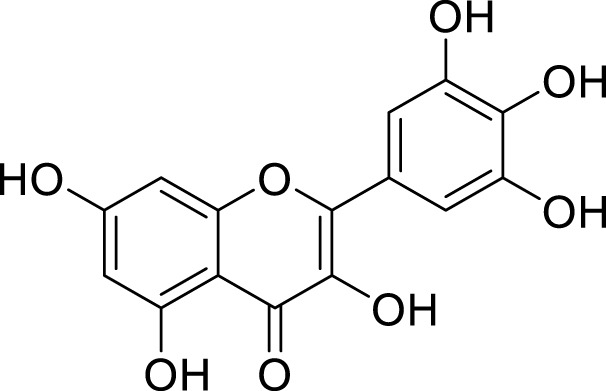	50 µM	MKN45 and MGC803 cells	CagA↓, NOX4↑, NRF2↓, SLC7A11↓, GPX 4↓, GSH↓, FLT3, Fe^2+^, MDA, and ROS↑	Myricetin regulated the inhibition of ferroptosis induced by *Helicobacter pylori* CagA through the NOX4/NRF2/GPX4 pathway	Lu et al. (2024)
	Amentoflavone (AF)	*Selaginella tamariscina* (P. Beauv.) Spring	C_30_H_18_O_10_	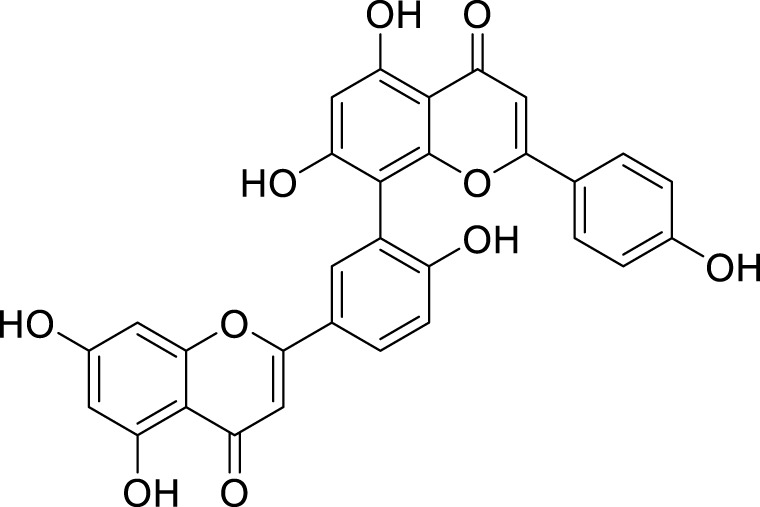	5μM, 24μM, 22μM, 40 μM	AGS and HGC-27 cells	miR-496↑, ATF 2↓, GPX4, SLC7A11, and GSH↓, Fe^2+^, ACSL4, MDA, and ROS↑	AF was able to suppress the proliferation and induce ferroptosis in AGS and HGC-27 cells through miR-496/ATF2 axis.	Tang et al. (2023)
Polysaccharides	Tremella fuciformis polysaccharides, (TFP)	*Tremella fuciformis* Berk	N/A	N/A	20μM, 40 μM	EBV-infected AGS and MNK45 cells	the mRNA levels of PTGS2 and Chac1↑, NRF2 and HO-1↓, Fe^2+^, MDA, and ROS↑, GPX4 xCT, and GSH↓	TFP induced ferroptosis in EBV-infected AGS and MNK45 cells cells by inhibiting NRF2/HO-1 signaling	Kong et al. (2024)
	Red ginseng polysaccharide (RGP)	*Panax ginseng* C. A. Mey	N/A	N/A	50μM, 100 μM	AGS	AQP3↓, p-PI3K and p-Akt↓, SLC7A11 and GPX4↓, ACSL4, Fe^2+^, MDA, LDH, and ROS↑	RGP promotes ferroptosis in AGS cells by inhibiting PI3K/Akt pathway through downregulation of AQP3	Wang et al. (2024c)
Quinonoids	Shikonin	*Lithospermum erythrorhizon* Siebold & Zucc	C_16_H_14_O_5_	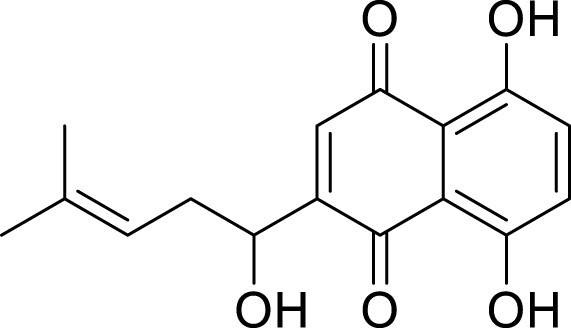	0.5μM, 1 μM 1.5μM, 2μM, 4μM, 10 μM	AGS and HGC-27	miR-9-3p↑, DLEU1↓, mTOR↓, ATF4, eIF2α, and the phosphorylation of eIF2α↑, SLC7A11↓, GSH↓, GPX4↓, Fe^2+^, MDA, and ROS↑	Shikonin may induce ferroptosis in AGS and HGC-27 cells through DLEU1/mTOR/GPX4 axis.	Wang et al. (2025c)
	Chrysophanol	*Rheum palmatum *L	C_15_H_10_O_4_	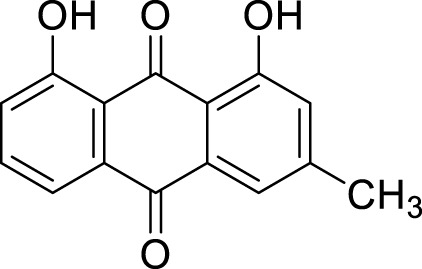	25μM, 50μM, 100 μM	HGC-27 and AGS	Bcl-2↓, Bax↑, cytochrome↑, mTOR, SLC7A11↓, GPX4↓, Fe^2+^↑, ROS↑	Chrysophanol may induce apoptosis and ferroptosis in HGC-27 and AGS cells by targeting and regulating mTOR.	Xi et al. (2024)
	Alkannin	*Lithospermum erythrorhizon* Siebold & Zucc	C_16_H_16_O_5_	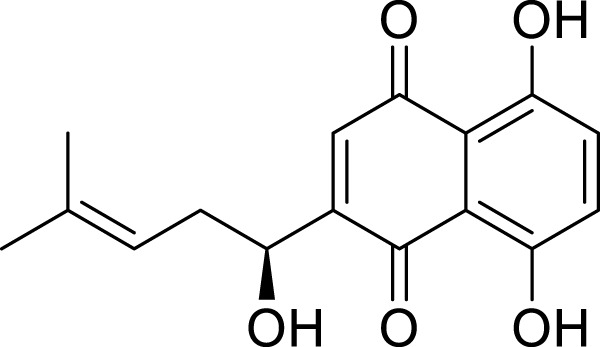	0.5μM, 1μM, 1.5 μM	AGS and HGC27 cells	c-Fos↓, SREBF1↓, Bax↑, Bcl-2↓, NRF2, SLC7A11, GPX4 and FTH1↓, Fe2+↑, MDA↑	Alkannin induces ferroptosis and apoptosis in AGS and HGC27 cells by inhibiting the c-Fos/SREBF1 signaling axis	Yu et al. (2025)
Alkaloids	Brucine	*Strychnos nux-vomica *L	C_23_H_26_N_2_O_4_	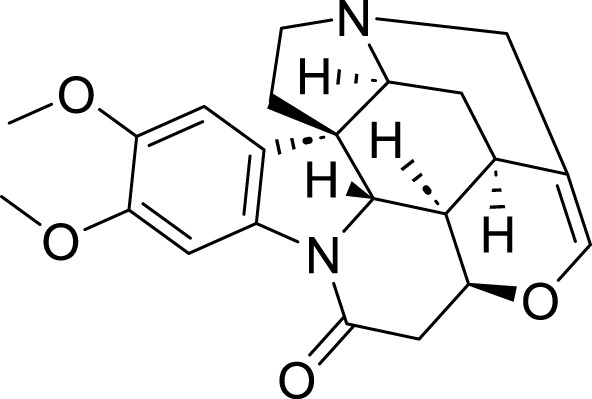	3.125μM, 6.25μM, 12.5μM, 25μM, 50μM, 100 μM 0.5μM, 1μM, 5μM, 10μM, 20 μM 30 μM	MKN45 and AGS cells	p53, ALOX12↑, SLC7A11↓, GSH↓, Fe^2+^, MDA, and ROS↑	Brucine regulates the p53/SLCA711/ALOX12 axis to cause ferroptosis in MKN45 and AGS cells.	Zhai et al. (2024)
					0.25μM, 0.5μM, 1 μM	AGS and HGC‐27 cells	NF‐κB signaling pathway↓, SLC7A11, GPX 4↓, NRF 2↓, ACSL 4↑, GSH↓, Fe2+↑, MDA↑, ROS↑	Brucine induces ferroptosis in AGS and HGC‐27 cells by indirectly inhibiting the NF-κB signaling pathway	Zhang et al. (2025a)
	Sanguinarine chloride (S.C)	*Sanguinaria canadensis*	C_20_H_14_ClNO_4_	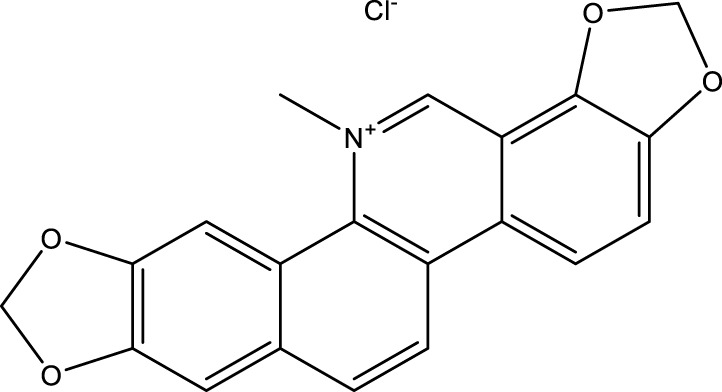	2μM, 4μM, 6 μM	MKN-45 cells	NOS2↑, SLC7A11↓, GPX4↓, GSH↓, Fe2+, MDA↑, ROS↑	S.C regulates SLC7A11/GPX4 through the SIRT1/NOS2/SOD1 signaling axis to induce ferroptosis in MKN-45 cells	Feng et al. (2025)
Others	Crocin	*Crocus sativus *L	C_44_H_64_O_24_	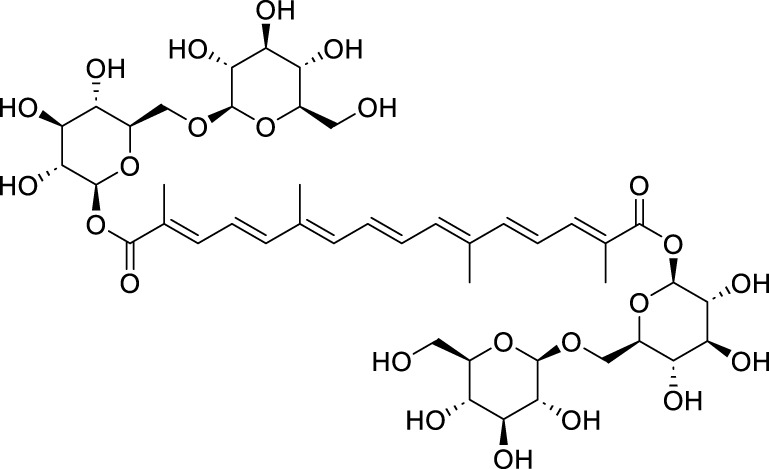	1mM, 3 mM	AGS, HGC27, and MKN45	NRF2↓, GGTLC2↓, GPX4, GSH, and lipid peroxidation levels↓, FACL4↑, Fe^2+^↑	Crocin may induces ferroptosis in GC cells through the NRF2/GGTLC2 pathway	Yan et al. (2025)
	Curcumin	*Curcuma longa *L	C_21_H_20_O_6_	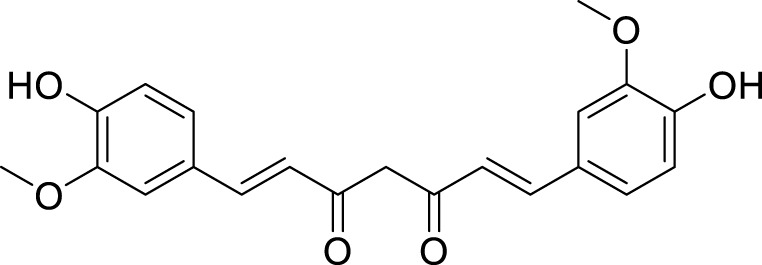	10 μM 20 μM	AGS and HGC-27	p-PI3K, p-AKT, and p-mTOR levels↓, SLC7A11, GPX4, and GSH↓, ACLS4, MDA, and ROS↑	Curcumin might suppress AGS and HGC-27 development by inducing autophagy-mediated ferroptosis by restraining the PI3K/AKT/mTOR signaling.	Zheng et al. (2025)
	Schizandrin A (Sch A)	*Schisandra chinensis *(Turcz.) Baill	C_24_H_32_O_6_	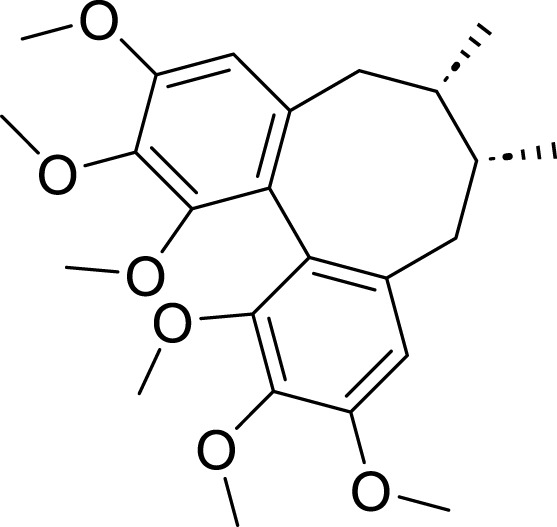	0.1µmol/, 1 μmol/L, 5 μmol/L, 25 μmol/L	AGS/5-Fu and SGC7901/5-Fu	TFRC↑, iron content↑, lipid peroxidation↑	The combination of Sch A and 5-Fu may induce the upregulation of TFRC, leading to ferroptosis in AGS/5-Fu and SGC7901/5-Fu cells	Hu et al. (2024)
Crude Extracts	Poria cocos	Poria cocos	N/A	N/A	50μM, 100 μM	SGC-7901	P53↑, GSH↑, SLC7A11↓, GPX4↓, ROS↑	Poria cocos inhibits the proliferation, migration, invasion, and EMT of SGC-7901 and GES-1 cells by activating ferroptosis.	Zheng et al. (2024)
	Mori Folium ethanol extracts(MFEE)	Mori Folium	N/A	N/A	30 μg/mL 60 g/mL 90 μg/mL	HGC27 and AZ521	HSP90/AKT axis↓, the phosphorylation levels of GSK3β↓, NRF2↓, GPX4↓, xCT↓, MDA, GSH/GSSG ratio↑, ROS↑	MFEE induces ferroptosis in gastric cancer cells HGC27 and AZ521 by inhibiting the AKT/GSK3β/NRF2 signaling axis	Hu et al. (2025)
	Chinese agarwood petroleum ether (CAPEE)	*Aquilaria sinensis* (Lour.) Spreng	N/A	N/A	4 μg·mL^−1^, 8 μg·mL^−1^	AGS, HGC27, and MGC803 cells	HO-1↑, lipid peroxide levels↑, Fe2+↑, ROS↑	CAPEE induces ferroptosis in AGS, HGC27, and MGC803 cell lines through the upregulation of HO-1	Ouyang et al. (2025)

ACSL4: acyl-CoA synthetase family member 4, ALOX12: arachidonate 12-lipoxygenase, Akt: protein kinase B, ALDH1A1:aldehyde dehydrogenase 1 family member A1, AQP3: aquaporin 3, ATF2: activating transcription factor 2, ATF4: activating transcription factor 4, ATG5: autophagy related 5, Bax: Bcl-2-associated X protein, Bcl-2: B-cell lymphoma/leukemia-2, CA: corosolic acid, CagA: Cytotoxin-associated gene A, CD44: cluster of differentiation 44, c-Fos: cellular proto-oncogene fos, CAPEE: Chinese agarwood petroleum ether, CHAC1: ChaC glutathione specific gamma-glutamylcyclotransferase 1, c-Myc: cellular myelocytomatosis oncogene, COX2: cyclooxygenase-2, DHA: dihydroartemisinin, DLEU1: deleted in lymphocytic leukemia 1, eIF2α: eukaryotic initiation factor 2 alpha subunit, FACL4: fatty acyl-CoA ligase 4, FLT3: Fms-like tyrosine kinase 3, FTH1: ferritin heavy chain 1, GGTLC2: gamma-glutamyltransferase light chain 2, GPX4: Glutathione peroxidase 4, GSH: glutathione, GSK3β: glycogen synthase kinase 3 beta, GSSG: oxidized glutathione, HO-1: heme oxygenase-1, HSP90: heat shock protein 90, KDM4D: lysine-specific demethylase 4D, Keap1: Kelch-like ECH-associated protein 1, LDH: lactate dehydrogenase, LC3B: Microtubule-associated protein 1 light chain 3 beta, MDA: malondialdehyde, mTOR: mammalian target of rapamycin, NCOA4: nuclear receptor coactivator 4, NF‐κB: Nuclear factor kappa-light-chain-enhancer of activated B cells, NRF2: Nuclear factor erythroid 2-related factor 2, NOS2: nitric oxide synthase 2, NOX1: NADPH oxidase 1, NOX4: NADPH oxidase 4, NQO1: NAD(P)H:quinone oxidoreductase 1, OCT3/4: octamer-binding transcription factor 3/4, PAH: Perillaldehyde, p-CaMKII: phosphorylated calcium/calmodulin-dependent protein kinase II, p-DRP1: phosphorylated dynamin-related protein 1, PDGFRB: platelet-derived growth factor receptor beta, p-GSK3β: phosphorylated glycogen synthase kinase 3β, PI3K: Phosphatidylinositol 3-Kinase, PTGS2: prostaglandin-endoperoxide synthase 2, ROS: reactive oxygen species, S.C: Sanguinarine chloride, SIRT1: Sirtuin 1, SLC1A5: solute carrier family 1 member 5, SLC7A11: solute carrier family 7 member 11, SOD1: superoxide dismutase 1, SREBF1: sterol regulatory element-binding factor 1, TFR1: transferrin receptor 1, TFRC: transferrin receptor, TOPK: tumor protein kinase, ULK1: Unc-51 like autophagy activating kinase 1

### 6.1 Saponins

The total saponins of Astragalus (TAS) represent the primary active component of *Astragalus membranaceus* (Fisch.) Bunge (Guo Z. et al., 2019). Previous studies have demonstrated that TAS possesses effective hepatoprotective, anti-inflammatory, and anti-fibrotic activities (Yongping et al., 2015; Zhou et al., 2016). The research conducted by Zou et al. (2025) indicates that TAS significantly enhances the expression of sirtuin 3 (SIRT3) in SGC7901 cells, leading to elevated levels of ROS, malondialdehyde (MDA), lactate dehydrogenase (LDH), and Fe^2+^. Concurrently, there is a reduction in the protein expressions of SLC7A11 and GPX4, along with an increase in the protein level of ACSL4. These changes—specifically the elevation of ROS, MDA, LDH, and Fe^2+^, alongside reduced SLC7A11/GPX4 and increased ACSL4—contribute to decreased cell proliferation and viability, ultimately inducing ferroptosis in GC cells. Similar to TAS, polyphyllin VII (PPVII), another bioactive compound from the rhizome of the traditional Chinese medicinal herb *Paris polyphylla* Sm, also exerts anti-GC effects by inducing ferroptosis (Thapa et al., 2022). It exhibits a broad spectrum of anti-cancer effects, including efficacy against lung cancer, liver cancer, and colorectal cancer (Ahmad et al., 2021). Xiang et al. (2023) reported that confirmed the ability of PPVII to induce ferroptosis in AGS and NCI-N87 GC cells. The underlying mechanism involves: reducing the inhibitory phosphorylation level of Unc-51 like autophagy activating kinase 1 (ULK1), decreasing the interaction between tumor protein kinase (TOPK) and ULK1, thereby activating the ULK1-dependent autophagic pathway. This pathway promotes the degradation of FTH1 through an autophagy-dependent mechanism, leading to downregulation of FTH1 levels. Consequently, this results in a substantial elevation in intracellular ROS, MDA, and free ferrous ions (Fe^2+^), ultimately inducing ferroptosis in GC cells. Polyphyllin I (PPI) is another natural compound derived from the rhizome of *P. polyphylla* Sm, known for its extensive antitumor properties (Yang et al., 2024). The study conducted by Zheng et al. (2023a) demonstrated that PPI can effectively inhibit the growth of GC cells. The authors hypothesized that the underlying mechanism involves the modulation of cell death-related microRNAs or interactions with antioxidant pathways, leading to alterations in the NRF2/FTH1 axis. This results in decreased intracellular levels of NRF2 and FTH1, which subsequently causes an increase in ROS, lipid peroxides, and ferrous ion concentrations within the cells. Ultimately, this process induces ferroptosis in AGS and MKN-45 cells, thereby exerting anti-tumor effects. This study is significant as it provides the first evidence that PPI induce ferroptosis in GC cells through regulation of the NRF2/FTH1 pathway to achieve their anti-tumor activity. In addition, Zheng et al. (2023b) also found that PPI can upregulate the expression of miR-124-3p in AGS and MKN-45 GC cells. This specific microRNA functions to downregulate the protein expression of NRF2 by directly targeting its 3′-untranslated region (UTR). The subsequent reduction in NRF2 levels alleviates its inhibitory effect on ferroptosis, resulting in increased intracellular ROS, lipid peroxides, and ferrous ions, ultimately leading to the induction of ferroptosis within GC cells. Therefore, PPI induces ferroptosis in these cells through modulation of the miR-124-3p/NRF2 signaling axis, thereby exerting a notable anti-tumor effect. Polyphyllin B (PB), a natural compound, is one of the active ingredients found in the traditional Chinese medicinal herb known as *P. polyphylla* Sm. It exerts its anti-tumor effects primarily by modulating immune responses and inducing apoptosis in cancer cells. PB is mainly used in the treatment of lung cancer, GC, and various other tumors (Wang Q. et al., 2024). The study conducted by Hu et al. (2023) revealed that PB enhances the transport of Fe^3+^ and facilitates the accumulation of intracellular Fe^2+^ through the regulation of several key proteins—specifically Microtubule-associated protein 1 light chain 3 beta (LC3B), TFR1, nuclear receptor coactivator 4 (NCOA4), and FTH1. Simultaneously, PB downregulates the expression of GPX4, thereby triggering ferroptosis in MKN-1 and NUGC-3 GC cells. This process simultaneously results in inhibited proliferation and invasion of GC cells, induced apoptosis, and disrupted cell cycle progression—all of which contribute to its anti-tumor effects. Ophiopogonins B (OP-B) is a bioactive component found in the roots of *Ophiopogon japonicus* (L. f.) Ker Gawl (Yuan F. et al., 2022). Recently, numerous studies have reported the anticancer effects of OP-B on various malignant tumors, including lung cancer, GC, and ovarian cancer (Zhang S. et al., 2021; Zhang et al., 2016; Yuan S. et al., 2022). Zhang et al. (2022) demonstrated that OP-B induces ferroptosis in AGS and NCI-N87 cells by inhibiting the GPX4/xC system. Asiaticoside (AC) is a triterpenoid derivative isolated from *Centella asiatica* (L.) Urb., known for its diverse pharmacological activities, including anti-inflammatory, antioxidant, antifibrotic, and neuroprotective effects (Bandopadhyay et al., 2023). AC significantly downregulates the levels of proteins associated with the Wnt/β-catenin signaling pathway [including β-catenin, phosphorylated glycogen synthase kinase 3β (p-GSK3β), glycogen synthase kinase 3 beta (GSK3β), cyclin D1, and cellular myelocytomatosis oncogene (c-Myc)] in AGS and HGC27 cells, as indicated by Ye et al. (2024) Concurrently, AC elevates intracellular Fe^2+^ and ROS levels while decreasing the expression of GPX4 and SLC7A11, thereby promoting ferroptosis. Furthermore, AC enhances the proportion of CD8^+^ T cells and reduces the expression of immune checkpoint molecules such as PD-L1, IFN-γ, and IL-10, effectively inhibiting mechanisms of immune evasion. These findings suggest that AC exerts its anti-gastric cancer effects by downregulating the Wnt/β-catenin pathway to induce ferroptosis while simultaneously suppressing immune escape pathways.

### 6.2 Terpenoids

Tanshinone IIA (Tan IIA), an active compound isolated from the roots of the traditional Chinese medicinal herb *Salvia miltiorrhiza* Bunge, exhibits a range of pharmacological activities, including cardiovascular protection (Han et al., 2008), anti-tumor effects (Wang et al., 2007), and antioxidant properties (Guo et al., 2018). The experiments conducted by Guan et al. (2020) demonstrated that tanshinone ⅡA could upregulate the expression levels of p53 in BGC-823 and NCI-H87 cells, specifically suppressing the expression of its target gene SLC7A11. This mechanism resulted in elevated intracellular levels of cysteine, GSH, and ROS, thereby triggering ferroptosis. Furthermore, the knockdown of p53 significantly inhibited the ferroptosis induced by tanshinone ⅡA. These findings provide compelling evidence that tanshinone ⅡA exerts its anti-tumor effects through the induction of ferroptosis. Subsequently, Ni et al. (2022) reported that tanshinone IIA suppresses the stemness of SGC-7901 and BGC-823 cells via an SLC7A11-dependent ferroptosis pathway. This suggests that tanshinone IIA may target the association between ferroptosis and cancer stem cells (CSCs), offering new insights for the development of GC therapies. Tanshinone I (Tan I) is another active extract derived from *S. miltiorrhiza* Bunge, known for its anti-inflammatory and antioxidant properties. Additionally, Tan I demonstrates anticancer activity against a variety of malignancies, including lung cancer, colorectal cancer, and cervical cancer (Ke et al., 2023). The study conducted by Xia et al. (2023) systematically confirms that Tan I exerts its growth-inhibitory effects on AGS and GC27 GC cells primarily through the induction of ferroptosis. The research identifies lysine-specific demethylase 4D (KDM4D) as a critical molecular target for the anticancer action of Tan I. Specifically, Tan I inhibits KDM4D, leading to an upregulation of p53 protein expression, which subsequently suppresses SLC7A11 gene expression and ultimately induces ferroptosis in GC cells. In summary, Tan I regulates ferroptosis by targeting the KDM4D/p53/SLC7A11 signaling axis, thereby achieving growth inhibition in GC cells. Pachymic acid (PA) is a naturally occurring triterpenoid compound and one of the principal active constituents of the traditional Chinese medicinal herb *Poria cocos* (PC) (Wei et al., 2022). It exhibits a variety of pharmacological activities, including anti-inflammatory, antioxidant, and hypoglycemic effects. Furthermore, it demonstrates antitumor properties against GC and pancreatic cancer (Xiao et al., 2024). The findings presented by Nie et al. (2024) provide preliminary evidence that PA induces ferroptosis in SGC-7901 and AGS cells through the modulation of the PDGFRB-mediated PI3K/Akt signaling pathway. This conclusion is supported by corresponding shifts in intracellular levels of MDA, Fe^2+^, GSH, and ROS, which collectively reflect the hallmark characteristics of ferroptosis. Curcumin (CUR) is one of the active compounds found in *Curcuma phaeocaulis* Valeton and *Curcuma longa* L., classified as a sesquiterpene with notable anticancer pharmacological properties (Sun et al., 2017). In addition to its anticancer effects, curcumin exhibits various other pharmacological activities, including anti-inflammatory, antioxidant, antiplatelet aggregation, and anticoagulant effects (Hashem et al., 2021). Feng et al. (2024) indicate that the combination treatment of CUR and cis–diamminodichloroplatinum (CDDP) can inhibit the expression of the antioxidant P62/KEAP1/NRF2 signaling pathway and its downstream protein NAD(P)H:quinone oxidoreductase 1 (NQO1) in drug-resistant GC cell lines MKN-45/DDP and AGS/DDP; consequently, this leads to significant alterations in intracellular levels of ROS, MDA, and iron ions associated with ferroptosis. Furthermore, it impacts the ferroptosis-related target GPX4, thereby inducing ferroptosis in drug-resistant GC cells. Ultimately, this results in a marked suppression of their proliferation, migration, and invasion capabilities. Dioscin is a natural compound derived from the Chinese herbal medicine *P. polyphylla* Sm. It has demonstrated anti-cancer effects against various types of cancer, including lung cancer, colorectal cancer, and prostate cancer. Lu et al. (2025) demonstrated that Dioscin exerts its effects on AGS and HGC-27 GC cells by downregulating the expression of SLC7A11 and GPX4. This mechanism results in insufficient synthesis of GSH alongside the accumulation of ROS and MDA, ultimately inducing ferroptosis. The findings suggest that Dioscin can induce ferroptosis in GC cells through modulation of the SLC7A11/GPX4 signaling axis, thereby further inhibiting metastasis in GC. Corosolic acid (CA), an active component derived from *Actinidia chinensis* Planch., can effectively inhibit the proliferation of various types of tumor cells and tumor growth. Lin et al. (2025) conducted experiments that demonstrated the potential of CA in significantly inhibiting the expression of Gpx4 when applied to AGS-CR cells; this intervention simultaneously induces ferroptosis and reduces the resistance of GC cells to cisplatin, thereby enhancing the anti-tumor efficacy of cisplatin. Perillaldehyde (PAH) is the primary component of *Perilla frutescens* (L.) Britton and exhibits a range of beneficial properties, including anti-inflammatory, antioxidant, anti-depressant, and anti-tumor effects (Zielińska-Błajet et al., 2021). Wang R. et al. (2025) identified that PAH induce ferroptosis in AGS and HGC27 cells through a dual mechanism. First, PAHs inhibit the system Xc^−^/GSH/GPX4 axis, leading to reduced cystine uptake and a subsequent decrease in GSH. This reduction impairs GPX4’s ability to eliminate lipid peroxides while simultaneously increasing Fe^2+^ levels, thereby triggering ferroptosis. Second, PAHs regulate the P62/Keap1/Nrf2 pathway, influencing the expression of proteins involved in iron transport and storage, which further modulates ferroptosis. Dihydroartemisinin (DHA) is a compound derived from the Medicine *Artemisia caruifolia* Buch.-Ham. ex Roxb. This compound exhibits multiple pharmacological effects, such as anti-inflammatory, anti-tumor, and antimalarial properties (Guo, 2016). Wang H. et al. (2025) demonstrated that DHA and DDP exhibit a synergistic effect in inducing ferroptosis in SGC-7901 and HGC-27 cells, by inhibiting the essential ferroptosis suppressor protein GPX4. The combined administration of these two agents effectively reduces both the expression and activity of GPX4, thereby preventing it from eliminating lipid peroxides and activating the ferroptosis pathway. This mechanism not only enhances the inhibitory effects on the proliferation, invasion, and migration of GC cells but also improves the efficacy of chemotherapy.

### 6.3 Flavonoids

Quercetin (Quer) is a naturally occurring flavonoid compound identified in various plant species, exhibiting multiple physiological activities. These activities encompass anti-inflammatory, antioxidant, antibacterial, and antiviral properties (Hosseini et al., 2021; Deepika and Maurya, 2022). Ding et al. (2024) found that Quer can inhibit the growth of AGS cells through the induction of ferroptosis. The underlying mechanism of this effect is closely related to the targeted regulation of solute carrier family 1 member 5 (SLC1A5). Specifically, Quer suppresses the activation of SLC1A5 in the NRF2/xCT pathway; consequently, this leads to reduced expression of GPX4—a key ferroptosis suppressor. Additionally, it inhibits the regulatory role of SLC1A5 on the p-Camk2/p-DRP1 pathway, thereby promoting lipid peroxidation. Furthermore, inhibition of SLC1A5 induces cellular iron accumulation and accelerates ferroptosis. This evidence underscores that Quer promotes ferroptosis in gastric cancer (GC) cells by specifically targeting SLC1A5 and modulating various downstream pathways. The study conducted by Huang J. et al. (2024) demonstrates that Quer significantly inhibits the viability of AGS and MKN45 cells *in vitro*. This process is mediated through the promotion of autophagy, as evidenced by the upregulation of autophagy-related proteins beclin1 and LC3B. Additionally, it is accompanied by corresponding changes in intracellular levels of GSH, MDA, and ROS. Furthermore, silencing ATG5 can reverse the aforementioned effects of quercetin, providing further evidence that its anti-GC activity is exerted via promoting autophagy-mediated ferroptosis. Baicalin, the active component derived from the Chinese medicinal herb *Scutellaria baicalensis* Georgi, exhibits pharmacological activities such as anti-inflammatory and antioxidant effects (Hu et al., 2021). In recent years, extensive research has been conducted on the anti-proliferative effects of baicalin across various malignant tumors at both cellular and molecular levels, encompassing liver cancer, colon cancer, and lung cancer (Singh et al., 2021; Zhao et al., 2021). Yuan et al. (2023) conducted a study that confirmed the ability of baicalin to induce the generation of ROS in AGS and SGC-7901 cells, thereby promoting ROS-dependent ferroptosis. This process enhances the sensitivity of GC cells to 5-fluorouracil (5-Fu) while inhibiting their resistance, effectively suppressing the occurrence and progression of GC. Shao et al. (2024) reported a significant ability of baicalin to enhance p53 expression in HGC27 and HGC27/L cells. This enhancement occurs through a p53-mediated pathway that inhibits SLC7A11 and GPX4, leading to the accumulation of ROS and subsequently activating ferroptosis. Furthermore, treatment with Pifithrin-α, a post-transcriptional inhibitor of p53, partially counteracts the effects of baicalin on SLC7A11 and GPX4. These findings confirm that baicalin induces ferroptosis via the p53-mediated SLC7A11/GPX4/ROS pathway. Importantly, this pathway may serve as a potential therapeutic target for reversing oxaliplatin resistance in GC, with baicalin identified as an effective intervention agent. Myricetin is a widely distributed flavonol that was first isolated from the bark of the *Morella rubra* Lour. It possesses various pharmacological activities, including anti-cancer, anti-allergic, antioxidant, and photoprotective effects (Gupta et al., 2020; Kumar et al., 2023). Lu et al. (2024) demonstrate that myricetin directly interacts with NADPH oxidase 4 (NOX4), maintaining its stability by inhibiting ubiquitin-mediated degradation. This interaction subsequently promotes the generation of ROS and induces ferroptosis in MKN45 and MGC803 cells. Additionally, myricetin can reverse the inhibitory effect of CagA on ferroptosis through the modulation of the NOX4/NRF2/GPX4 pathway, revealing its potential to exert anti-GC effects via the induction of ferroptosis. The Amentoflavone (AF) is a natural biflavonoid compound that was isolated from the *Selaginella tamariscina* (P. Beauv.) Spring over 40 years ago (Xiong et al., 2021). AF exhibits a variety of biological activities, including anti-angiogenic, antibacterial, antiviral, and anti-inflammatory properties (Cai et al., 2019; Zhang et al., 2014; Hwang et al., 2013; Coulerie et al., 2013). Tang et al. (2023) found that treatment with AF can enhance the expression levels of miR-496 in AGS and HGC-27 cells. Furthermore, miR-496 exerts its effects by targeting activating transcription factor 2 (ATF2), which is significantly upregulated in GC tissues and cells, thereby inhibiting the proliferation of GC cells and inducing ferroptosis. These findings suggest that AF promotes ferroptosis via the modulation of the miR-496/ATF2 axis, thus contributing to its anti-GC effects.

### 6.4 Polysaccharides

Tremella polysaccharide (TFP) is a type of polysaccharide extracted from *Tremella fuciformis* Berk., which is recognized as the primary active component responsible for its nutritional and health benefits (Fu et al., 2021). TFP exhibits anti-inflammatory, antioxidant, and blood sugar-lowering properties (hui et al., 2013; Ruan et al., 2018). The research conducted by Kong et al. (2024) indicates that TFP significantly diminishes the migratory capacity of EBV-infected AGS and MKN45 cells, while concurrently upregulating the mRNA expression levels of prostaglandin-endoperoxide synthase 2 (PTGS2) and ChaC glutathione-specific gamma-glutamylcyclotransferase 1 (Chac1). Additionally, TFP downregulates the protein expressions of NRF2, HO-1, GPX4, and xCT by inhibiting the NRF2/HO-1 signaling pathway. Furthermore, overexpression of NRF2 can counteract the downregulatory effects of TFP on GPX4 and xCT expression. This corroborates that TFP induces ferroptosis in EBV-infected gastric cancer cells through inhibition of the NRF2/HO-1 signaling pathway. Red ginseng polysaccharides (RGP) are one of the active components of *Panax ginseng* C. A. Mey (Wang et al., 2016). Previous studies have demonstrated that RGP possesses significant immunostimulatory and regulatory effects, making it a natural compound with potential therapeutic value in anti-tumor treatments (Guo et al., 2022). Wang Y. et al. (2024) conducted a study demonstrating that RGP exhibit antitumor effects. The underlying mechanism involves RGP downregulating the expression of aquaporin 3 (AQP3), which simultaneously inhibits the activity of the PI3K/Akt signaling pathway and promotes ferroptosis in AGS cells, ultimately leading to a reduction in cell proliferation and viability.

### 6.5 Quinonoids

Shikonin is the primary bioactive component extracted from the roots of the traditional Chinese medicinal herb, *Lithospermum erythrorhizon* Siebold & Zucc. (Boulos et al., 2019). Recent studies have demonstrated that shikonin possesses a variety of biological activities, including antitumor, anti-inflammatory properties, and the ability to promote wound healing (Guo C. et al., 2019). The study conducted by Wang Y. et al. (2025) indicates that shikonin exhibits anti-GC effects through the induction of ferroptosis. Oridonin is capable of reducing the expression of the oncogene DLEU1 in AGS and HGC27 cells, with a mechanism potentially linked to the inhibition of the deleted in lymphocytic leukemia 1 (DLEU1)/mammalian target of rapamycin (mTOR) pathway, leading to decreased levels of GPX4. Furthermore, DLEU1 has been shown to interact with miR-9-3p. This finding corroborates the notion that the DLEU1/mTOR/GPX4 axis may be pivotal in oridonin-induced ferroptosis in GC cells. Additionally, it suggests that DLEU1 has the potential to serve as a promising therapeutic target for GC treatment. Chrysophanol is a naturally occurring anthraquinone compound, initially isolated as the principal active component from the Chinese herb medicinal *Rheum palmatum* L. (Prateeksha et al., 2019). Research indicates that chrysophanol exhibits a diverse array of pharmacological activities, including anti-cancer, antiviral, anti-diabetic, and anti-inflammatory effects. Furthermore, it demonstrates antiprotozoal properties as well as lipid-lowering capabilities, in addition to hepatoprotective and neuroprotective functions (Xie et al., 2019). Xi et al. (2024) demonstrate that chrysophanol increases the levels of ROS, total iron, and Fe^2+^ in HGC-27 and AGS cells in a dose-dependent manner. This effect may induce ferroptosis by targeting the regulation of the mTOR pathway, as suggested by GEO screening results. The ferroptosis inhibitor ferrostatin-1 can mitigate its impact on cell viability and the expression of SLC7A11 and GPX4. Furthermore, overexpression of mTOR can reverse this inhibitory effect, providing further support for its role in inducing ferroptosis through mTOR modulation. Additionally, this process is accompanied by dose-dependent apoptosis, disruption of mitochondrial membrane potential, and release of cytochrome c. Alkannin is a quinone compound derived from the roots of *L. erythrorhizon* Siebold & Zucc. It exhibits various pharmacological effects, including anti-inflammatory, antioxidant, and anticancer properties. Yu et al.'s research (Yu et al., 2025) demonstrated that Alkannin induces ferroptosis in GC cells through the inhibition of the cellular proto-oncogene fos(c-Fos)/sterol regulatory element-binding factor 1(SREBF1) signaling axis. Specifically, Alkannin downregulates the expression of c-Fos, which directly binds to the SREBF1 promoter to activate its transcription. SREBF1 is essential for maintaining intracellular lipid homeostasis by promoting the expression of genes involved in lipid synthesis, thereby inhibiting ferroptosis. When Alkannin inhibits c-Fos, it disrupts the transcriptional activation of SREBF1, leading to disturbances in lipid and redox homeostasis and consequently alleviating the inhibition of ferroptosis. This process ultimately results in ferroptosis induction within GC cells. Concurrently, this mechanism also initiates apoptosis; together, these two processes collaborate to exert an anti-gastric cancer effect.

### 6.6 Alkaloids

Brucine is a weakly basic indole alkaloid and one of the primary bioactive and toxic components found in *Strychnos nux-vomica* L. (Noman et al., 2022). Modern pharmacological research and clinical practice have demonstrated that brucine possesses a wide range of pharmacological activities, including anti-tumor, anti-inflammatory, analgesic effects, as well as actions on the cardiovascular and nervous systems (Lu et al., 2020). The research conducted by Zhai et al. (2024) indicates that brucine can upregulate the expression levels of p53 and ALOX12 in AGS and MKN45 cells while inhibiting the expression of SLC7A11, thereby inducing ferroptosis. Furthermore, silencing p53 was found to reverse these effects, confirming the critical role of p53 in this process. Collectively, these findings suggest that brucine induces ferroptosis in GC cells through the regulation of the p53/SLC7A11/ALOX12 signaling axis, providing a potential intervention strategy for GC treatment. Furthermore, Zhang B. et al. (2025) conducted research demonstrating that brucine induces ferroptosis in AGS and HGC-27 cells by indirectly inhibiting the nuclear factor kappa-light-chain-enhancer of activated B cells NOX4 (NF-κB) signaling pathway. The mechanism is as follows: Brucine can synergize with the ferroptosis inducer Erastin to significantly enhance intracellular levels of iron ions, MDA, and ROS, while concurrently reducing GSH levels, thus triggering ferroptosis. Moreover, the effect of Brucine on ferroptosis is closely associated with its inhibition of the NF-κB pathway. Experimental evidence suggests that this inhibitory action on the NF-κB pathway represents a potential mechanism through which Brucine induces ferroptosis and exerts its anti-gastric cancer effects. S.C. is an alkaloid derived from *Sanguinaria canadensis* and has been demonstrated to function as a multi-faceted anti-cancer agent (Peng et al., 2025). Feng et al. (2025) found that S.C regulates SLC7A11/GPX4 through the SIRT1/NOS2/SOD1 signaling axis to induce ferroptosis in MKN-45 cells. The specific mechanism is as follows: S.C interacts with GC cells and modulates the activity of the deacetylase SIRT1. This modulation influences the expression of nitric oxide synthase 2 (NOS2) by altering its acetylation levels. Subsequently, NOS2 downregulates the expression of SLC7A11 and GPX4 via regulation of superoxide dismutase 1 (SOD1). During this process, the downregulation of SLC7A11 leads to reduced cystine uptake, while decreased levels of GPX4 and inhibition of GSH result in an accumulation of intracellular ROS and MDA, ultimately triggering ferroptosis.

### 6.7 Others

Crocin is the primary bioactive component of *Crocus sativus* L. (Pourmousavi et al., 2024; Hashemzaei et al., 2020), classified as a water-soluble carotenoid. It possesses significant anti-inflammatory, antioxidant, and antitumor properties. Yan et al. (2025) reported that crocin can facilitate the translocation of Nrf2 protein from the nucleus to the cytoplasm. Nrf2 has the ability to directly bind to the promoter region of gamma-glutamyltransferase light chain 2 (GGTLC2), thereby inhibiting its transcription and expression. This mechanism subsequently attenuates apoptosis and ferroptosis in AGS, HGC27, and MKN45 cell lines. In summary, crocin induces ferroptosis in GC cells by regulating the Nrf2/GGTLC2 signaling pathway. Curcumin, a principal bioactive compound characterized by its yellow or orange pigments, is derived from the rhizome of the perennial herb *C. longa* L., belonging to the ginger family (Unlu et al., 2025). Curcumin exhibits a range of pharmacological properties, including antimicrobial, anti-inflammatory, antioxidant, and immunomodulatory effects. Given that inflammation is one of the contributing factors to cancer development, research indicates that curcumin may be beneficial in both the prevention and treatment of cancer (Devassy et al., 2015). Zheng et al. (2025) found that curcumin can induce autophagy and inhibit the PI3K/AKT/mTOR signaling pathway in AGS and HGC-27 cells. Based on these findings, it can be inferred that curcumin may suppress the activation of the PI3K/AKT/mTOR pathway, thereby triggering autophagy-mediated ferroptosis and ultimately inhibiting the progression of GC. The bioactive lignan compound Schizandrin (Sch A), isolated from *Schisandra chinensis* (Turcz.) Baill., exhibits a range of biological properties, including anti-inflammatory, hepatoprotective, antitumor, and antioxidant effects (Zhu et al., 2021; Fu et al., 2022). The experiments conducted by Hu et al. (2024) have preliminarily elucidated that the combination of Sch A and 5-FU can overcome the resistance of AGS/5-Fu and SGC7901/5-Fu cells to 5-FU. The underlying mechanism may involve the upregulation of transferrin receptor (TFRC) expression induced by the combined treatment. Specifically, this upregulation promotes excessive accumulation of intracellular iron content and lipid peroxidation levels. Ultimately, this process induces ferroptosis in 5-FU-resistant GC cells, thereby reversing drug resistance.

### 6.8 Crude extracts

Poria cocos is employed in Chinese herbal medicine for its diuretic properties and renal protective effects. Furthermore, it plays a significant role in the modulation of immune system functions (Guo et al., 2025). Zheng et al. (2024) demonstrated that poria cocos can enhence the expression of ferroptosis-related genes GPX4 and SLC7A11, while simultaneously increasing cellular ROS levels. Notably, the co-culture with ferroptosis inhibitors can reverse this effect. These findings suggest that Poria cocos inhibits the proliferation, migration, invasion, and epithelial-mesenchymal transition (EMT) of SGC-7901 cells through the activation of ferroptosis, thereby offering a novel therapeutic strategy for GC treatment. Mulberry leaves, derived from the mulberry tree, are a Chinese herb medicinal (Jeong et al., 2017). They possess various pharmacological activities, including blood glucose reduction, blood pressure lowering, anti-inflammatory effects, antioxidant properties, and antitumor capabilities (Joh et al., 2015; Jeong et al., 2016; Lyu et al., 2021). The study conducted by Hu et al. (2025) reveals that the ethanol extract of mulberry leaves (MFEE) exerts a potent anti-GC effect by inhibiting the AKT/GSK3β/NRF2 axis, leading to downregulation of xCT and GPX4, which induces ferroptosis in HGC27 and AZ521 cells. These findings underscore the potential of MFEE as an effective strategy or adjunctive agent for GC treatment. *Aquilaria sinensis* (Lour.) Spreng. is a Chinese herbal medicine known for its anti-tumor properties. Research indicates that Chinese agarwood petroleum ether extract (CAPEE) demonstrate significant cytotoxic effects against various cancer cell lines, including those associated with colorectal and pancreatic cancers (Hashim et al., 2016). Ouyang et al. (2025) conducted research demonstrating that CAPEE induces ferroptosis in GC cell lines AGS, HGC27, and MGC803 by up-regulating HO-1 (heme oxygenase-1). Specifically, this process is characterized by increased levels of intracellular Fe^2+^, lipid peroxides, and ROS. The knockdown of HO-1 diminishes the sensitivity of GC cells to CAPEE and inhibits the ferroptosis it triggers. Furthermore, CAPEE promotes G0/G1 phase arrest through the DNA damage-p21-cyclin D1/CDK4 axis and mediates apoptosis via the BAX/BAK pathway, thereby collectively impeding the progression of GC.

## 7 Traditional Chinese medicine formulas prevent and treat GC by regulating ferroptosis

**FIGURE 5:**
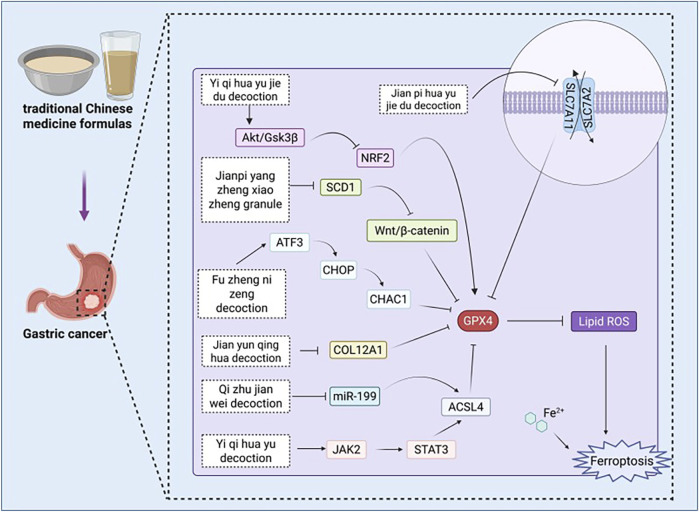
Pharmacological mechanisms of traditional Chinese medicine formulas targeting ferroptosis in the treatment of gastric cancer. ACSL4: acyl-CoA synthetase family member 4, Akt: protein kinase B, ATF3: activating transcription factor 3, CHAC1: Chac glutathione specific gamma-glutamylcyclotransferase 1, CHOP: C/EBP homologous protein, COL12A1: collagen type XII alpha 1 chain, GPX4: Glutathione peroxidase 4, GSH: glutathione, GSK3β: glycogen synthase kinase 3 beta, GSSG: oxidized glutathione, JAK2: Janus Kinase 2, MDA: malondialdehyde, ROS: reactive oxygen species, SCD1: Stearoyl-CoA Desaturase 1, SLC7A11: solute carrier family 7 member 11, STAT3:Signal transducer and activator of transcription 3.

The active components of traditional Chinese medicine formulas exert anti-cancer effects through the synergistic action of multiple constituents. Among these, the induction of ferroptosis in GC cells represents one of the key pathways to achieve this therapeutic effect. Currently, as the role of ferroptosis in the regulation of GC progression is gradually being elucidated, exploring the specific mechanisms by which traditional Chinese medicine formulas combat GC—particularly their relationship with the ferroptosis pathway—has become an important research direction and a hot topic in the field of GC prevention and treatment (Figure 5; Table 2).

**TABLE 2:** Pharmacological mechanisms of traditional Chinese medicine formulas targeting ferroptosis in the treatment of gastric cancer.

Herbal formula	Ingredients	Cell lines or animal type	Main indicators	Mechanism	References
Qi zhu jian wei decoction (QZJWD)	*Astagalus membranaceus* (Fisch.) *Beg.var.mongholicus* (Bge.) Hsiao, *Polygonatum odoratum* (Mill.) Druce, *Atractylodis macrocephala* Koidz., *Curcuma phaeocaulis* Val., *Evodia rutaecarpa* (Juss.) Benth, *Coptis chinensis* Franch., *Agrimonia Pilosa* Ledeb., 30g *Rabdosia rubescens* (Hemsl.) Hara, *Glycyrrhiza uralensis* Fisch., and *Salvia chinensis* Benth	AGS and HGC-27 cells	miR-199-3p↓, MDA, ROS ACSL4, and Fe^2+^↑, GPx↓	QZJWD promotes ferroptosis in AGS and HGC-27 cells through the exosome-mediated miR-199-3p/ACSL4 signaling pathway	Dai et al. (2025)
Jian pi yang zheng xiao zheng granule (JPYZXZ)	*Astragalus membranaceus* (Fisch.) Bunge, *Codonopsis pilosula* (Franch.) Nannf., *Atractylodes macrocephala* Koidz., *Angelica sinensis* (Oliv.) Diels, *Paeonia lactiflora* Pall., *Sparganium stoloniferum* (Buch.-Ham. ex Graebn.) Buch.-Ham. ex Juz., *Curcuma zedoaria* (Christm.) Roscoe, *Vladimiria souliei* (Franch.), *Citrus reticulata* Blanco, *Scleromitrion diffusum* (Willd.) R. J. Wang, *Salvia chinensis* Benth., and *Glycyrrhiza uralensis* Fisch	HGC-27 and MKN-45 cells	SCD1↓, Wnt/β-catenin↓, GPX4↓, xCT↓, MDA, ROS, and Fe^2+^↑, GSH↓	The JPYZXZ promotes lipid metabolism and induces ferroptosis in HGC-27 and MKN-45 cells by reshaping the SCD1/Wnt/β-catenin signaling axis	Wang et al. (2025d)
Jian yun qing hua decoction (JYQHD)	*Pseudostellaria heterophylla* (Miq.) Pax, *Astragalus membranaceus* (Fisch.) Bunge, *Atractylodes macrocephala* Koidz., *Dioscorea polystachya* Turcz. Coix lacrymajobi L., *Salvia miltiorrhiza* Bge., *Curcuma phaeocaulis* Valeton, Citrus aurantium L., Bupleurum chinense DC.	SGC-7901 and MKN-45 cells	COL12A1↓, ROS, MDA, and Fe^2+^↑, GSH↓	JYQHD induces ferroptosis in SGC-7901 and MKN-45 by downregulating COL12A1	Liu et al. (2023b)
Yi qi hua yu decoction (YQHY)	*Astragalus membranaceus* (Fisch.) Bunge, *Codonopsis pilosula* (Franch.) Nannf., *Citrus reticulata* Blanco, *Pinellia ternata* (Thunb.) Ten. ex Breitenb., *Atractylodes macrocephala* Koidz.,Paeonia lactiflora Pall.,Angelica sinensis (Oliv.) Diels, Sparganium stoloni erum, Buch. -Ham.,Curcuma phaeocaulis Valeton, Scleromitrion diffusum (Willd.) R. J. Wang, Salvia chinensis Benth.,Poria cocos(Schw.)Wolf, Vladimiria souliei (Franch.), Amomum villosum Lour., and Glycyrrhiza uralensis Fisch	AGS cells	JAK2, STAT3↑, ACSL4↑, GSH↓, ROS, MDA, and Fe^2+^↑	YQHY can induce ferroptosis in AGS cells by affecting the JAK2/STAT3 pathway and the expression of ACSL4	Song et al. (2022)
Yi qi hua yu jie du decoction (YJD)	*Astragalus membranaceus* (Fisch.) Bunge, *Codonopsis pilosula* (Franch.) Nannf., *Citrus reticulata* Blanco, *Pinellia ternata* (Thunb.) Ten. ex Breitenb., *Poria cocos* (Schw.)Wolf, *Aucklandia costus* Falc., *Amomum villosum* Lour., *Atractylodes macrocephala* Koidz., *Paeonia lactiflora* Pall., *Angelica sinensis* (Oliv.) Diels, *Sparganium stoloniferum* (Buch.-Ham. ex Graebn.) Buch.-Ham. ex Juz., *Curcuma phaeocaulis* Valeton, *Salvia chinensis* Benth., *Scleromitrion diffusum* (Willd.) R. J. Wang, and *Glycyrrhiza uralensis* Fisch	AGS/DDP and MKN-45/DDP cells	p-Akt↓, p-GSK3β↓, NRF2↓, GPX4↓, GSH/GSSG↓, ROS, MDA, and Fe^2+^↑	YJD can promote ferroptosis in cisplatin-resistant gastric cancer cells AGS/DDP and MKN-45/DDP through the Akt/GSK3β/NRF2/GPX4 signaling pathway	Huang et al. (2024b)
Jian pi hua yu jie du decoction (JHJD)	*Astragalus membranaceus* (Fisch.) Bunge, *Wolfiporia cocos* (F.A. Wolf) Ryvarden & Gilb., Pseudostellaria heterophylla (Miq.) Paxt, *Scleromitrion diffusum* (Willd.) R.J.Wang, *Hericium erinaceus* (Bull.) Pers., *Curcuma phaeocaulis* Valeton, *Atractylodes macrocephala* Koidz., *Panax notoginseng* (Burkill) F.H.Chen,and *Gecko gecko* Linnaeus, 1758	GPL C57BL/6 mice	GPX4↓, SLC7A11↑, ALOX15↓, GSSG↓, GSH↑, Fe^2+^↓	JHJD alleviates GPL by regulating key factors such as SLC7A11 and GPX4 to inhibit ferroptosis	Zhang et al. (2025b)
Fu zheng ni zeng decoction (FZNZ)	*Astragalus membranaceus* (Fisch.) Bunge, *Atractylodes macrocephala* Koidz., *Glycyrrhiza uralensis* Fisch., *Citrus reticulata* Blanco, *Cyperus rotundus* L., *Panax notoginseng* (Burkill) F. H. Chen ex C. H. Chow, *Pinellia ternata* (Thunb.) Ten. ex Breitenb., *Poria cocos* (Schw.) Wolf, *Curcuma aromatica* Salisb., *Salvia miltiorrhiza* Bunge, *Actinidia chinensis* Planch., and *Scutellaria barbata* D. Don, *Solanum lyratum* Thunb	MC cells	ATF3, CHOP↑, CHAC1↑,GPX4↓, GSH↓, ROS, MDA, and Fe^2+^↑	FZNZ, centered on ATF3/CHOP/CHAC1, regulates glutathione metabolism and induces endoplasmic reticulum stress, collectively facilitating the occurrence of ferroptosis in MC cells	Chu et al. (2022)

ACSL4: acyl-CoA synthetase family member 4, Akt: protein kinase B, ALOX15: arachidonate 15-lipoxygenase, ATF3: activating transcription factor 3, CHAC1: Chac glutathione specific gamma-glutamylcyclotransferase 1, CHOP: C/EBP homologous protein, COL12A1: collagen type XII alpha 1 chain, GPX4: Glutathione peroxidase 4, GSH: glutathione, GSK3β: glycogen synthase kinase 3 beta, GSSG: oxidized glutathione, FZNZ: Fuzheng Nizeng Decoction, JAK2: Janus Kinase 2, JHJD: Jian pi hua yu jie du decoction, JPYZXZ: Jian pi yang zheng xiao zheng granule, JYQHD: Jian yun qing hua decoction, MDA: malondialdehyde, mTOR: mammalian target of rapamycin, QZJWD: Qi zhu jian wei decoction, YJD: Yi qi hua yu jie du decoction, ROS: reactive oxygen species, SCD1: Stearoyl-CoA Desaturase 1, SLC7A11(xCT): solute carrier family 7 member 11, STAT3: Signal transducer and activator of transcription 3, YQHY: Yi qi hua yu decoction.

The Qi zhu jian wei decoction (QZJWD) composed of the following ingredients: *Astagalus membranaceus*(Fisch.)Beg.*var.mongholicus*(Bge.) Hsiao, *Polygonatum odoratum* (Mill.) Druce, *Atractylodis macrocephala* Koidz., *C. phaeocaulis* Val., *Evodia rutaecarpa* (Juss.) Benth, *Coptis chinensis* Franch., *Agrimonia Pilosa* Ledeb.,30g *Rabdosia rubescens* (Hemsl.) Hara, *Glycyrrhiza uralensis* Fisch., and *Salvia chinensis* Benth. The research conducted by Dai et al. (2025) indicates that miR-199-3p inhibits ferroptosis in GC cells HGC-27 and AGS by targeting ACSL4. Furthermore, QZJWD significantly downregulates the expression of miR-199-3p in exosomes, thereby relieving its inhibitory effect on ferroptosis. In addition, QZJWD can also regulate indicators associated with ferroptosis, resulting in increased levels of MDA, ACSL4, ROS, and Fe^2+^ within the cells while decreasing GPx levels, thus promoting ferroptosis in GC cells. In summary, QZJWD modulates ferroptosis through the exosome-mediated miR-199-3p/ACSL4 signaling pathway and exerts an anti-gastric cancer effect.

The Jian pi yang zheng xiao zheng granule (JPYZXZ) is composed of various herbal ingredients, including *Astragalus membranaceus* (Fisch.) Bunge, *Codonopsis pilosula* (Franch.) Nannf., *Atractylodes macrocephala* Koidz., *Angelica sinensis* (Oliv.) Diels, *Paeonia lactiflora* Pall., *Sparganium stoloniferum* (Buch.-Ham. ex Graebn.) Buch.-Ham. ex Juz., *Curcuma zedoaria* (Christm.) Roscoe, *Vladimiria souliei* (Franch.), *Citrus reticulata* Blanco, *Scleromitrion diffusum* (Willd.) R. J. Wang, *Salvia chinensis* Benth., and *G. uralensis* Fisch. The study by Wang X. et al. (2025) indicates that JPYZXZ downregulates the expression of SCD1, producing effects comparable to either the knockout of SCD1 or the application of the inhibitor A939572. Following the downregulation of SCD1, the Wnt/β-catenin signaling pathway is inhibited, concurrently negatively regulating GPX4, xCT, FASN, and ACC1. This cascade results in a reduction in TC, TG, and lipid droplet numbers. These changes lead to an increase in ROS, Fe^2+^, and MDA levels while decreasing GSH levels, thereby inducing ferroptosis. In summary, JPYZXZ promotes lipid metabolism and induces ferroptosis by reshaping the SCD1/Wnt/β-catenin signaling axis, ultimately inhibiting the progression of low-adhesion gastric cancer cells (PCC).

The composition of Jian yun qing hua decoction (JYQHD) includes *Pseudostellaria heterophylla* (Miq.) Pax,Astragalus membranaceus (Fisch.) Bunge, Atractylodes macrocephala Koidz., Dioscorea polystachya Turcz., Coix lacrymajobi L., Salvia miltiorrhiza Bge., Curcuma phaeocaulis Valeton, Citrus aurantium L., Bupleurum chinense DC. The study conducted by Liu B. et al. (2023) indicates that JYQHD can effectively reduce the intracellular GSH levels in MKN-45 and SGC-7901 cells, while simultaneously increasing ROS, MDA, and Fe^2+^ levels, thereby inducing ferroptosis. Furthermore, JYQHD downregulates the expression of collagen type XII Alpha 1 chain (COL12A1), alleviating its inhibitory effect on ferroptosis. The research confirms that JYQHD induces ferroptosis through the regulation of COL12A1, thereby exerting an anti-gastric cancer effect.

Yi qi hua yu decoction (YQHY) is composed of the following ingredients: *Astragalus membranaceus* (Fisch.) Bunge, *C. pilosula* (Franch.) Nannf.,*C. reticulata* Blanco, *Pinellia ternata* (Thunb.) Ten. ex Breitenb.,*Atractylodes macrocephala* Koidz.,*P. lactiflora* Pall.,*Angelica sinensis* (Oliv.) Diels, *Sparganium stoloni erum,* Buch. -Ham.,*C. phaeocaulis* Valeton, *S. diffusum* (Willd.) R. J. Wang, *Salvia chinensis* Benth.,*Poria cocos*(Schw.)Wolf, *V. souliei* (Franch.),and *Amomum villosum* Lour. and Glycyrrhiza uralensis Fisch. The study conducted by Song et al. (2022) demonstrated through network pharmacology and experimental validation that YQHY, with quercetin as its primary active component, targets TP53 and ACSL4, thereby inducing ferroptosis in GC cells via the Janus Kinase 2 (JAK2)/signal transducer and activator of transcription 3 (STAT3) pathway. Experimental evidence indicates that YQHY significantly increases the levels of MDA in AGS cells while concurrently decreasing GSH levels. Furthermore, it regulates the activity of the JAK2/STAT3 pathway and the expression of ACSL4, contributing to the induction of ferroptosis. These mechanisms may represent critical pathways through which YQHY exerts its anti-recurrence and anti-metastatic effects against GC.

The Yi Qi Hua Yu Jie Du decoction (YJD) composed of the following ingredients: *Astragalus membranaceus* (Fisch.) Bunge, *C. pilosula* (Franch.) Nannf., *C. reticulata* Blanco, *P. ternata* (Thunb.) Ten. ex Breitenb., *Poria cocos*(Schw.)Wolf, *Aucklandia costus* Falc., *A. villosum* Lour., *Atractylodes macrocephala* Koidz., *P. lactiflora* Pall., *Angelica sinensis* (Oliv.) Diels, *S. stoloniferum* (Buch.-Ham. ex Graebn.) Buch.-Ham. ex Juz., *C. phaeocaulis* Valeton, *Salvia chinensis* Benth., *S. diffusum* (Willd.) R. J. Wang, and *G. uralensis* Fisch. Research conducted by Huang W. et al. (2024) indicates that YJD inhibits the activation of the Akt/GSK3β pathway in cisplatin-resistant GC cells, specifically AGS/DDP and MKN-45/DDP, leading to a reduction in nuclear NRF2 levels and subsequently suppressing GPX4 expression. Following intervention, there was a significant increase in intracellular levels of ROS, MDA, and Fe^2+^, while the GSH/GSSG ratio decreased. This series of changes induced ferroptosis in the cells. These findings suggest that YJD can promote ferroptosis in cisplatin-resistant GC cells through the Akt/GSK3β/NRF2/GPX4 signaling pathway, thereby enhancing their sensitivity to cisplatin treatment.

Jian pi hua yu jie du decoction (JHJD) is composed of the following nine traditional Chinese medicinal herbs: *Astragalus membranaceus* (Fisch.) Bunge, *Wolfiporia cocos* (F.A. Wolf) Ryvarden & Gilb., Pseudostellaria heterophylla (Miq.) Paxt, *S. diffusum* (Willd.) R.J.Wang, *Hericium erinaceus* (Bull.) Pers., Curcuma phaeocaulis Valeton, *Atractylodes macrocephala* Koidz., *Panax notoginseng* (Burkill) F.H.Chen,and *Gecko gecko* Linnaeus, 1758. The research conducted by Zhang S. et al. (2025) indicates that JHJD regulates the relationship between *H. pylori* infection and MNU-induced gastric precancerous lesions (GPL) in a mouse model, promoting an increase in GSH concentration within GPL while simultaneously reducing the levels of GSSG and Fe^2+^. This process rebalances the SLC7A11/GPX4 signaling pathway, thereby inhibiting lipid peroxidation and ferroptosis, ultimately effectively curbing the progression of GPL.

The Fu zheng ni zeng decoction (FZNZ) is composed of the following ingredients: *Astragalus membranaceus* (Fisch.) Bunge, *Atractylodes macrocephala* Koidz., *G. uralensis* Fisch., *C. reticulata* Blanco, *Cyperus rotundus* L., *P. notoginseng* (Burkill) F. H. Chen ex C. H. Chow, *P. ternata* (Thunb.) Ten. ex Breitenb., *Poria cocos*(Schw.)Wolf, *Curcuma aromatica* Salisb., *S. miltiorrhiza* Bunge, *A. chinensis* Planch., *Scutellaria barbata* D. Don, as well as *Solanum lyratum* Thunb. The study conducted by Chu et al. (2022) revealed that FZNZ increases the levels of Fe^2+^, ROS, and MDA in MC cells while simultaneously decreasing the content of GSH and GPX4. Furthermore, FZNZ induces endoplasmic reticulum (ER) stress, leading to the upregulation of ATF3, C/EBP homologous protein (CHOP), and chac glutathione specific gamma-glutamylcyclotransferase 1 (CHAC1), thereby promoting ferroptosis. Consequently, it is hypothesized that FZNZ acts through a core mechanism involving ATF3/CHOP/CHAC1 to regulate glutathione metabolism and induce ER stress, collectively facilitating ferroptosis in MC cells and contributing to its anti-gastric cancer effects.

## 8 Discussion

This study conducted a systematic review of the role of Chinese herbal medicine, encompassing both the traditional Chinese medicine extracts and traditional Chinese medicine formulas in the treatment of GC through the modulation of ferroptosis mechanisms. Theoretically, this study represents the first comprehensive examination of the relationship among “Chinese herbal medicine - Ferroptosis - Gastric Cancer,” integrating disparate basic research evidence from recent years. It elucidates the core targets (such as GPX4, ACSL4, Nrf2, SLC7A11, etc.) and key pathways through which Chinese herbal medicine regulates ferroptosis. This work addresses a critical gap in understanding the mechanistic connections between the “multi-target effects of Chinese herbal medicine” and the “precise regulation of ferroptosis.” From the perspective of application significance, this research offers novel insights into the clinical treatment of GC and the development of new pharmacological agents. On one hand, existing studies have confirmed that Chinese herbal medicine can enhance the sensitivity of GC cells to chemotherapy drugs (such as cisplatin) by inducing ferroptosis. This provides experimental evidence supporting the clinical strategy of “combining Chinese herbal medicine with chemotherapy” to improve efficacy while reducing toxicity. On the other hand, the components of Chinese herbal medicine identified in this study that possess ferroptosis-regulatory activity (such as curcumin and quercetin) may serve as lead compounds for developing new anti-cancer drugs, thereby establishing a solid foundation for creating novel therapeutics with well-defined constituents and clear targets.

Additionally, we summarized the key anticancer chemical groups of certain active components found in traditional Chinese medicine extracts. The saponins contain multifunctional hydroxyl groups, glycosidic bonds, and ester groups that can interact with target proteins to modulate their activity while enhancing solubility and membrane permeability. The terpenoids possess carbonyls, epoxides, and carboxyl/hydroxyl groups that regulate ROS, inhibit signaling pathways, and reduce activity through binding to targets. Flavonoids feature ortho-dihydroxy phenolic hydroxyls, carbonyls, and methoxy groups capable of chelating Fe^2+^, increasing hydrophobic interactions, and improving cellular penetration. Quinones exhibit a quinone structure along with hydroxyl groups that can generate ROS, covalently bind to targets, and elevate Fe^2+^ levels. Alkaloids possess quaternary amine nitrogen atoms as well as ester/methoxy functionalities that facilitate electrostatic interactions and hydrophobic effects for pathway regulation. Among other compounds, polyene structures are able to chelate Fe^2+^ while polysaccharide repeating units can modulate signaling pathways. These functional groups induce ferroptosis in GC cells by regulating iron metabolism, inhibiting antioxidant systems, and modulating signaling pathways.

Although this study systematically reviewed the potential pharmacological mechanisms of Chinese herbal medicine in treating GC through the regulation of ferroptosis, several fundamental limitations and challenges persist in translating experimental research into clinical practice. Firstly, the majority of current research remains limited to *in vitro* cell experiments or animal models. The former struggles to accurately simulate the heterogeneity of human GC and its tumor microenvironment, while the latter exhibits differences from the physiological and pathological states present in humans. Consequently, this may result in where the “ferroptosis of GC cells induced by Chinese herbal medicine,” as observed in basic research, cannot be fully replicated in clinical patients. Meanwhile, the majority of studies have not performed systematic preclinical pharmacokinetic and toxicological assessments. For example, the absorption, distribution, metabolism, and excretion profiles of active components in Chinese herbal medicine remain poorly understood. Furthermore, critical data regarding liver and kidney toxicity at effective anti-cancer doses, as well as long-term safety associated with continuous administration, are insufficiently documented. These gaps represent significant barriers to advancing these therapies into phase I clinical trials. Secondly, the complexity of Chinese herbal medicine has intensified the barriers in the process of transformation. Whether it is traditional Chinese medicine extracts or traditional Chinese medicine formulas, their component systems exhibit a high degree of complexity. This makes it difficult to precisely identify the 1 to 2 key components that play a core role, and it is also challenging to clarify the specific interaction between these components and the key targets of the ferroptosis pathway, such as GPX4, ACSL4, and Nrf2. This situation may not fully align with the modern clinical drug development requirements of “clear components and clear targets”, and poses challenges for subsequent clinical dose standardization and the assessment of therapeutic stability. Moreover, the ferroptosis mechanism is complex and not yet fully elucidated. The regulation of key pathways (such as GPX4, SLC7A11, p53, etc.) by Traditional Chinese medicine extracts often involves intricate interactions among multiple targets. Additionally, the potential connections between ferroptosis signaling pathways and other forms of regulated cell death, the identification of core execution molecules, as well as their specific functions in physiological processes still require further clarification. Finally, ferroptosis is not a cell death modality exclusive to tumor cells; normal tissues may also sustain damage due to the activation of this pathway. However, current research primarily concentrates on the induction of ferroptosis in GC cells through Chinese herbal medicine, without systematically evaluating its safety profiles for normal gastric mucosal cells and vital organs. This raises potential safety concerns regarding the phenomenon of “inhibiting cancer while harming normal tissues” in clinical applications. Such issues significantly constrain their further promotion and application in clinical settings.

Based on the core findings of this study and the current state of research in the field, future investigations should be advanced in the following directions to facilitate the translation of basic research on ferroptosis regulation by Chinese herbal medicine into clinical GC treatment: In light of the current research predominantly concentrating on cellular and animal models, it is imperative to establish a research framework that more closely aligns with clinical practice in the future. On one hand, systematic preclinical pharmacokinetic and toxicological studies must be conducted: pharmacokinetic studies to elucidate the absorption, distribution, metabolism, and excretion (ADME) profiles of active components in Chinese herbal medicine *in vivo*, simultaneously conducting toxicological studies to assess potential hepatotoxicity, nephrotoxicity, and long-term safety at effective doses. On the other hand, human tumor xenograft models (PDX) and organoid models ought to be employed to validate the anti-cancer efficacy of Chinese herbal medicine under conditions that replicate the microenvironment of human GC. This approach will provide critical data necessary for advancing to phase I clinical trials. Furthermore, it is essential to conduct a more in-depth analysis of the “multi-target synergy mechanism” by which Chinese herbal medicine regulates ferroptosis. This includes investigating the interactions between the components of Chinese herbal medicine and ferroptosis pathways, as well as their crosstalk with other PCD pathways such as apoptosis and pyroptosis.

## 9 Conclusion

In summary, there is an urgent need to conduct in-depth investigations into specific biomarkers associated with ferroptosis and to elucidate the potential regulatory molecular mechanisms involved. Although existing studies have indicated that Chinese herbal medicine possesses potential in exerting anti-GC effects through the modulation of ferroptosis, however, further research is necessary to systematically elucidate their target mechanisms and associated signaling pathways. Identifying specific ferroptosis inducers and inhibitors derived from Chinese herbal medicine not only aids in elucidating their mechanisms of action but also provides a theoretical foundation for the development of more targeted and effective therapeutic strategies. As basic research and clinical translation continue to advance, the application of ferroptosis regulatory mechanisms in clinical practice for disease prevention and treatment is becoming increasingly feasible.

## Data Availability

The original contributions presented in the study are included in the article/supplementary material, further inquiries can be directed to the corresponding author.
