# Correction: Pharmacological mechanisms of traditional Chinese medicine in treating gastric cancer: a focus on the ferroptosis regulation

**DOI:** 10.3389/fphar.2026.1918992

**Published:** 2026-07-10

**Authors:** Shanshan Gao, Huijuan Wang

**Affiliations:** 1 First Clinical Medical College, Shandong University of Traditional Chinese Medicine, Jinan, China; 2 Experimental Center, Shandong University of Traditional Chinese Medicine, Jinan, China

**Keywords:** gastric cancer, ferroptosis, traditional Chinese medicine extracts, traditional Chinese medicine formulas, pharmacological mechanism

Affiliation 2 Experimental Center, Shandong University of Traditional Chinese Medicine, Jinan, China was erroneously given as First College of Clinical Medicine, Shandong University of Traditional Chinese Medicine, Jinan, China.

The original version of this article has been updated.

